# ZrCo_3_B_2_ and HfCo_3_B_2_ Electrodes Go on Stage for Oxygen Evolution Reaction

**DOI:** 10.1002/advs.77373

**Published:** 2026-09-16

**Authors:** Fatma Aras, Ulrich Burkhardt, Simone G. Altendorf, Yuri Grin, Iryna Antonyshyn

**Affiliations:** ^1^ Max‐Planck‐Institut für Chemische Physik fester Stoffe Dresden Germany; ^2^ Fritz‐Haber‐Institut der Max‐Planck‐Gesellschaft Berlin Germany

**Keywords:** chemical behavior, chemical bonding, electrocatalyst, intermetallic compounds, oxygen evolution reaction

## Abstract

The water electrolysis is one of the key processes for producing hydrogen—a clean and sustainable energy carrier. However, its wide application onto the industrial scale still lack of the long‐lasting and highly efficient electrocatalysts for the kinetically sluggish oxygen evolution reaction (OER). Here, we demonstrate the OER performance of intermetallic precursors *TM*Co_3_B_2_ (*TM* = Zr, Hf) in the bulk state allowing to avoid the detrimental effects of binders and/or supports and assess the intrinsic activity and stability of electrode material. Significantly lower OER overpotentials for *TM*Co_3_B_2_ (ca. 270 mV) compared to elemental Co (ca. 320 mV), almost doubled current density values at maximum potential of 1.95 V vs. RHE (0.7 A cm^−2^ for ZrCo_3_B_2_ and 1.1 A cm^−2^ for HfCo_3_B_2_) as well as the stability of their OER activity for almost 500 h reaction period bring forward those intermetallic compounds among other known electrocatalysts. Furthermore, our results prominently illustrate the importance of crystal structures and chemical bonding of intermetallic compounds, remarkably influencing the OER performance. These highly OER active and stable ZrCo_3_B_2_ and HfCo_3_B_2_ electrodes will pave the way for usage of intermetallic compounds in the large‐scale electrolyzer applications.

## Graphical abstract

The intermetallic compounds ZrCo_3_B_2_ and HfCo_3_B_2_ with distinctive chemical bonding features are excellent OER precursors, restructuring into OER‐active electrocatalysts delivering significantly high OER activities compared to the elemental Co, which is obvious from their lower onset potentials and increased obtained current densities. The stability measurements for almost 500 h at elevated current densities support their long‐lasting OER performances at harsh reaction conditions.

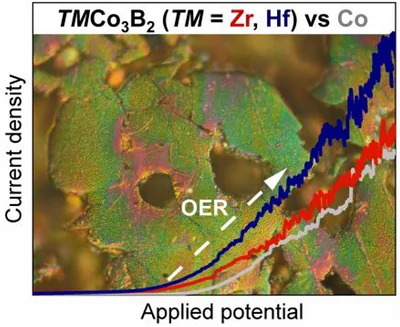

## 1 Introduction

The development of eco‐friendly energy technologies is essential to deal with the rising worldwide energy usage [1, 2]. Water electrolysis powered by renewable energy sources has great potential to provide a solution to this ever‐increasing energy demand. However, the efficiency of water electrolysis should be improved by developing new electrocatalysts for the anodic half‐reaction, namely oxygen evolution reaction (OER). The rational design of OER electrocatalysts should compromise such electrode properties as activity, the long‐term stability of activity, electrical conductivity, resistance against bulk corrosion, and low production cost [3]. Among noble metal‐free OER electrocatalysts in alkaline media, cobalt‐based ones have been explicitly put forward in many studies because of their remarkable OER activity and stability [3, 4, 5, 6, 7, 8, 9, 10, 11, 12]. The spinel‐type transition metal oxides such as Co_3_O_4_ [4], NiCo_2_O_4_ [4], perovskite oxides [13], as well as more sophisticated OER catalysts such as Co_3_O_4_ nanocubes on thin CoO layer [14], mesoporous CoNiFe oxide [15] and [(V_2_O_3_‐CoFe_2_O_4_)@C/NF] [16], in addition to the OER electrocatalysts listed in Table S1 and corresponding references, are only few examples of Co‐containing efficient OER electrocatalysts in an alkaline medium. The structural changes of cobalt oxide catalysts before and/or during OER to the electroactive oxo species of cobalt have been extensively studied in order to elucidate the mechanism of this dynamic transformation [17]. The formation of the OER‐active species on the electrode surface for efficient OER is an important phenomenon governing the OER activity and stability of the electrode materials, and the pristine material restructuring under OER toward the in situ formation of the OER‐active species needs to be elucidated for further rational development of the OER electrocatalysts. The profound understanding of the reaction mechanism as well as disentangling of the different effects onto the OER activity of the electrocatalyst and its stability under reaction conditions is a multifaceted and complex task. However, intermetallic compounds offer a great platform to investigate the transformation of precursors to the actual OER electrocatalysts [18, 19, 20]. They possess a well‐defined crystal structure (definite atomic arrangement on the surface), unique electronic structure (due to the combination of two or more elements in a specific way), particular chemical bonding (defining the stability of materials as well as state of the surface under reaction conditions), and sufficient electrical conductivity (allowing to obtain electrocatalytically active dynamic layer on top of conductive bulk electrode). Combination of these advantages with the detailed analysis of electrochemical and chemical behavior under reaction conditions make us one step closer to the knowledge‐based development and optimization of the desired catalyst characteristics. Furthermore, only with profound understanding of the electrocatalyst structure and its changes under reaction conditions, the purpose‐oriented choice and/or development of electrocatalyst scalability route can be considered.

In the shed of the aforementioned OER‐active Co‐based materials reported in the literature, intermetallic compounds of Co as supposed active center in combination with *TM* (Zr, Hf) and B were considered as materials of choice for this study. On one hand, boron‐containing intermetallic compounds are characterized by their good electrical conductivity, outstanding hardness and wear resistance (especially, the borides with refractory metals) [21], which can be advantageous for the material integrity in the electrochemical environment. In addition, the OER studies on cobalt borides (Co_2_B with OER onset potentials of 1.61 V vs. RHE (reference hydrogen electrode) [22], Co_3_B with onset potential of 1.55 V vs. RHE [23]) explicitly indicate the improvement in the electrocatalytic activities, on which boron potentially decreases the energetic and kinetic barrier of the formation of electroactive species [22]. Furthermore, the Fe and Ni substitutions in Co_2_B, such as (Ni_0.75_Co_0.25_)_2_B [24], (Co_0.9_Ni_0.1_)_2_B [25], (Co_0.7_Fe_0.3_)_2_B [26], provided a synergistic effect of Fe and Ni on the OER activity of Co_2_B electrodes. On the other hand, both hafnium and zirconium show noticeable corrosion resistance to bases [21], which allows to consider these elements as valuable counterpart of stable OER electrocatalysts. For instance, Co/Ni‐substituted zirconium and hafnium diborides have shown reasonable activities toward OER, e.g. overpotential at 10 mA cm^−2^ is 350 mV for Zr_0.8_Ni_0.2_B_2_ and 370 mV for Zr_0.8_Co_0.2_B_2_ [27], and it was found as 320 mV for Hf_0.8_Ni_0.2_B_2_ and 340 mV for Hf_0.8_Co_0.2_B_2_ [28]. Furthermore, recently, the ternary compound Hf_2_B_2‐2_ *_x_* Ir_5+_ *_x_* was discovered as a promising precursor for OER in proton exchange membrane (PEM) electrolysis [29]. The partial oxidation of the surface and formation of catalytically active layer is controlled by the structural and chemical bonding features of the initial Hf_2_B_2‐2_ *_x_* Ir_5+_ *_x_* compound, possessing the cage‐like structure of a covalently bonded Ir‐B anionic framework, hosting the positively charged Hf atoms [30]. Therefore, looking for iridium‐free materials with similar structural characteristics combined with the above‐mentioned choice of constituent elements was selected as one possible way for development of new water splitting electrocatalysts.

The ZrCo_3_B_2_ and HfCo_3_B_2_ ternary intermetallic compounds have been investigated in this study as OER electrodes in 1 M KOH electrolyte using several electrochemical techniques. The initial characterization of the pristine materials accompanied by the chemical bonding analysis as well as the ex situ characterization of the surface and the underlying bulk electrode after OER provided insight into the chemical behavior of the ZrCo_3_B_2_ and HfCo_3_B_2_ compounds under OER conditions. The latter is key for the interpretation of the OER performance.

## 2 Results and Discussion

In the ternary systems of early transition metals (*TM*) with cobalt—as an analog of iridium—and boron, compositionally similar phases were found around 17 at.% of *TM* [31]. Two different crystal structures were debated in the literature: a hexagonal one of the structure type CeCo_3_B_2_ (space group *P*6/*mmm*, later mentioned as *TM*Co_3_B_2_.*h*) [32], or a rhombohedral one of the structure type ZrCo_3_B_2_ (space group *R* $\bar{3}$, later—*TM*Co_3_B_2_.*r*) [33]. The phase relationship between both is still under discussion. In both structural motifs, boron and cobalt atoms form 3D‐framework. While in *TM*Co_3_B_2_.*h* boron atoms are far apart from each other (the B‐B distance is ca. 2.8 Å), in the *TM*Co_3_B_2_.*r*, the formation of boron dumbbells with a distance of ca. 1.7 Å was reported [33]. This was the reason to focus first on the rhombohedral modification. Also, the electron backscatter diffraction (EBSD) of the pristine *TM*Co_3_B_2_ samples indicates the rhombohedral structure dominates in the investigated materials (particularly in the case of ZrCo_3_B_2_, Figure S1).

Combining the specific chemical characteristics of each selected elements into their intermetallic compounds with (at least partially) ordered crystal and unique electronic structures [18], ZrCo_3_B_2_ and HfCo_3_B_2_ were chosen to be investigated as advantageous precursor materials for OER in alkaline medium. Our aim is not only to achieve exceptional activity and durability of these materials for OER, but also to disclose the influence of chemical interactions between the elements in the studied materials onto their chemical properties under harsh electrochemical conditions, and consequent dynamic surface chemistry determining key performance indicators of these compounds as OER electrocatalysts. With this respect, quantum chemical bonding analysis of ZrCo_3_B_2_ and HfCo_3_B_2_ has been performed to shed light on the contribution of atomic interaction as well as the electronic state of the constituent elements onto the chemical behavior of studied electrodes being decisive for their OER performance.

The chemical bonding analysis of rhombohedral *TM*Co_3_B_2_.*r* was performed applying the electron localizability approach—a quantum chemical technique in position space [34]. Topological analysis of the electron density using the Quantum Theory of Atoms in Molecules (QTAIM [35]) allowed to evaluate atomic shapes and effective charges in position space (Figure S2). While the QTAIM *TM* atoms reveal close to spherical shapes characteristic for the ionic or strongly polar species, the cobalt and boron QTAIM atoms show rather plane faces toward Co and B neighbors, characteristic rather for covalent interactions [34]. Integration of electron density within the QTAIM shapes yields that both—cobalt and boron—carry small negative effective charges between −0.2 and −0.4. The *TM* species reveal rather large positive charges between +1.4 and +1.7, which are on the level of the values usually obtained for more electropositive earth‐alkaline (cf. +1.3 – +1.5 for Sr in Sr_8_Si_46_ [36]) and rare‐earth [37] (cf. +1.6 for Y in YGa_6_ [38]) metals. The QTAIM charges indicate already essential ionicity of atomic interactions.

**FIGURE 1:**
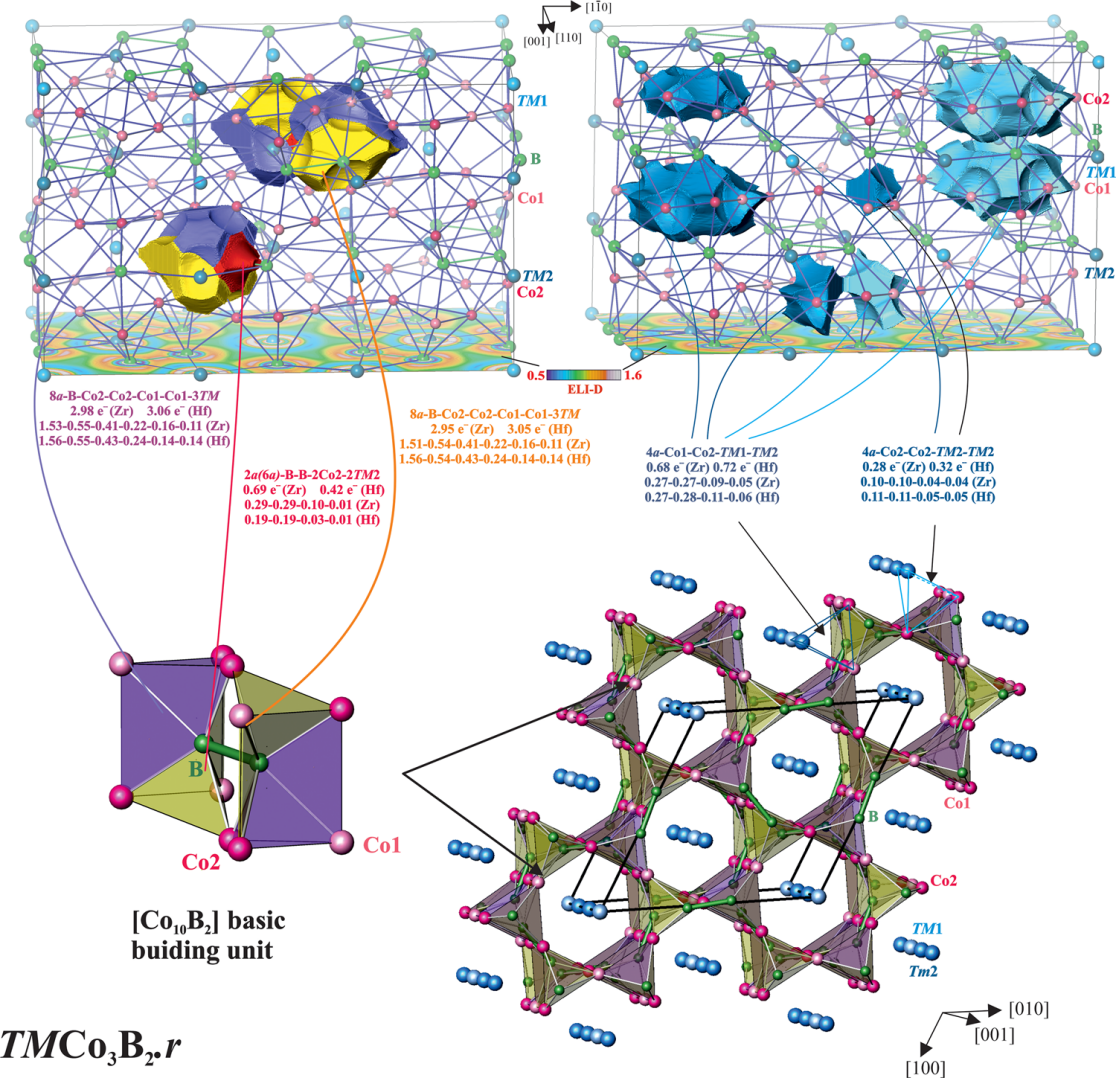
Chemical bonding in *TM*Co_3_B_2_.*r* (*TM* = Zr, Hf) as obtained from the electron localizability approach: (*top left*) Localization of ELI‐D maxima around boron and cobalt atoms in the (001) plane (*upper part of the panel*) and basins of three types of bonds – 8‐atomic (*violet* and *orange*) as well as mostly two‐atomic B‐B (*red*)—which form the basic building block of the polyanion [Co_10_B_2_] (*bottom left*). (*top right*) Two types of polar four‐atomic Co_2_ *TM*_2_ bonds in the tubular cavities of the framework. For each bond, its participating atoms (*first line*), total bond population for Zr‐ and Hf‐containing compounds (*second line*), and atomic contributions for the ZrCo_3_B_2_.*r* (*third line*) and HfCo_3_B_2_.*r* compound (*fourth line*) are listed (*bottom right*) General bonding picture revealing covalently bonded polyanionic 3D Co‐B framework with the tubular cavities occupied by cationic Zr (Hf) species.

The analysis of bonding was continued employing a combination of the electron density with the electron localizability indicator (ELI‐D [34]). The distribution of ELI‐D in the valence region in *TM*Co_3_B_2_.*r* is characterized by strong maxima in vicinity of the B‐B and B‐Co contacts and low local maxima in the region between the Co and *TM* (Figure 1, *top*). The first group of ELI‐D maxima visualizes the formation of the anionic framework in the structure, mainly realized by the two‐atomic 2*a*‐B‐B bonds and multiatomic (8‐atomic) interactions where the boron atoms make the largest contributions to the bond basin populations (Figure 1, *top left*). The cobalt atoms of the basic building units [Co_10_B_2_] are shared among the neighboring units, so that the total composition is achieved *TM*(Co1)_4/4_(Co2)_6/3_B_2_ = *TM*Co_3_B_2_.

The interaction between the cationic *TM* species and the anionic [Co_10_B_2_] framework is realized in form of two types of four‐atomic bonds ‒ 4*a*‐Co1Co2*TM*1*TM*2 and 4*a*‐(Co2)_2_(*TM*2)_2_. The space around *TM*1 is filled symmetrically by (twelve) basins of the first type, while the surrounding of *TM*2 contains 6 basins of each type (Figure 1, *top right*). All 4*a*‐bonds are polar: cobalt atoms contribute the main part of the basin populations.

The general picture shows weakly anisotropic bonding in the *TM*Co_3_B_2_.*r* compounds: the 3D anionic framework is constructed by covalently interlinked [Co_10_B_2_] basic structure units formed by 8*a*‐BCo_4_ *TM*_3_ and Co‐ and *TM*‐supported 2*a*‐B‐B bonds. The framework bears tubular cavities along [001], where the cationic *TM* are located by polar four‐atomic *TM*_2_Co_2_ bonds. In this regard, the covalently interlinked [Co_10_B_2_] units providing general stability (or at least controlled limited surface oxidation of Co) are considered as the precursor for in situ formed thin layers of Co oxide/(oxy)hydroxide electroactive species. On the other hand, the channels with Zr (Hf) are accessible for OH^−^ ions, and their oxidation leads to the enhanced surface area. In general, the role of Zr and Hf atoms in the catalytic activity and stability of the electrode materials is supposed to be indirect. Compared to the elemental Co (with zero charge), the Co atoms in the *TM*Co_3_B_2_ structures have small negative charges (cf. above), which are a result of electronic effect of the neighboring Hf and B atoms. Therefore, the distinguished reactivity of the Co atoms is strongly influenced by the synergistic effect of the other constituent elements in the intermetallic compounds. Moreover, in the crystal structure of *TM*Co_3_B_2_, the Zr and Hf are placed in the tubular cavities along [001] axis, formed by the framework of covalently interlinked [Co_10_B_2_] basic structure units. Zr and Hf play both roles. On one hand, they support covalent bonding in the [Co_10_B_2_] polyanions, on another one, due to the cationic character (cf. QTAIM results), they support the surface “cleaning” by oxidation and migration to the electrolyte. There are not experimental facts indicating participation of Zr and Hf in the catalytic cycle itself.

**FIGURE 2:**
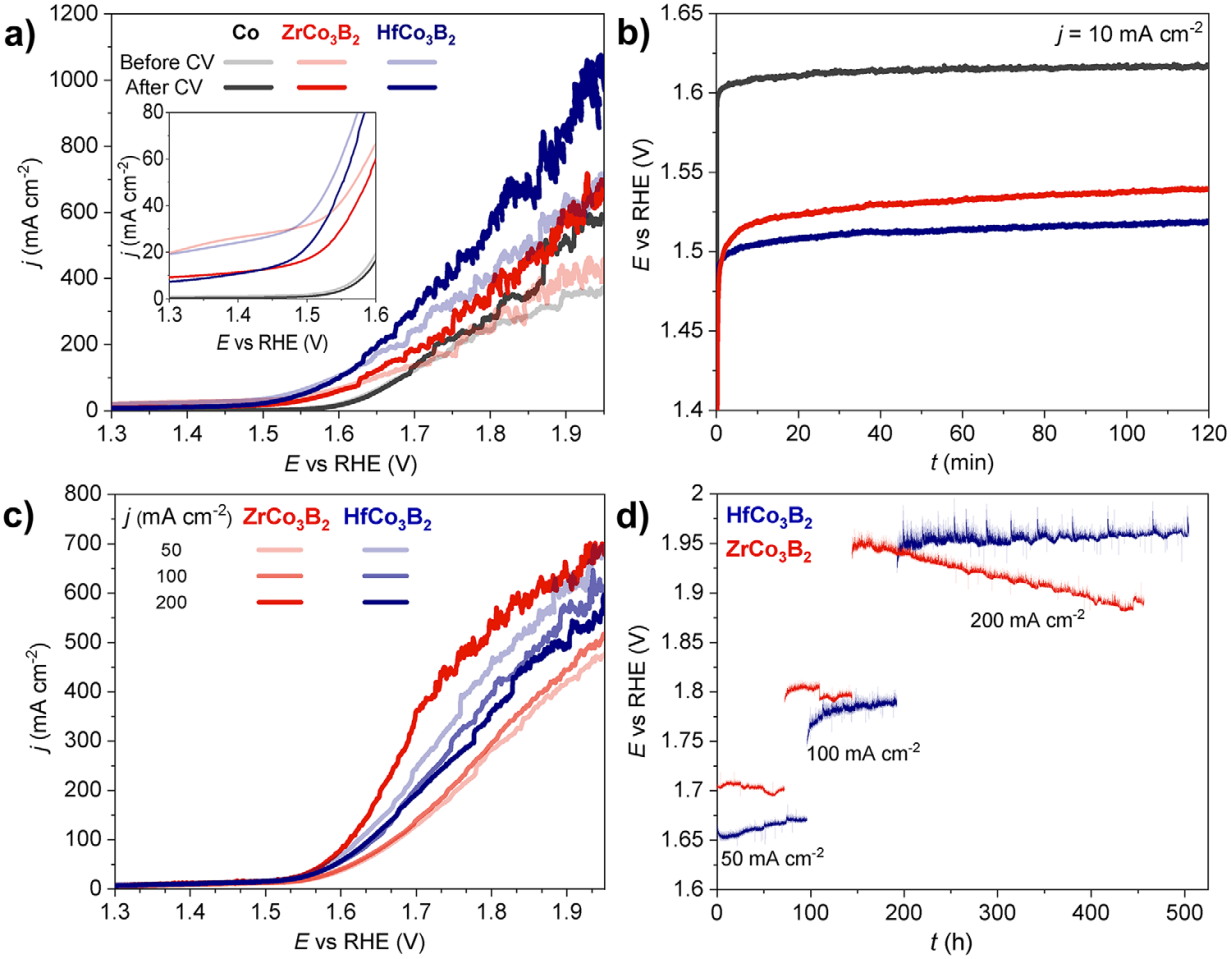
OER performance of ZrCo_3_B_2_ (*red*) and HfCo_3_B_2_ (*navy*): (a) OER activity before and after CV pre‐treatment, (b) *st*‐CP (*j* = 10 mA cm^−2^, *t* = 2 h), (c) LSVs after each step of *lt*‐CP, and (d) *lt*‐CP at elevated current densities of 50, 100, and 200 mA cm^−2^.

Together with the enlightenment of the chemical bonding analysis, the comprehensive characterization of the pristine materials plays a decisive role for further understanding of their chemical behavior under reaction conditions. The success of *TM*Co_3_B_2_ synthesis was revealed by the powder x‐ray diffraction (PXRD, Figure S3, lattice parameters a = 8.4269(2) Å, c = 9.1280(3) Å for ZrCo_3_B_2_.*r* and *a* = 8.3815(2) Å, *c* = 9.1140(3) Å for HfCo_3_B_2_.*r* being in good agreement with the literature data [33]) and scanning electron microscopy (SEM, Figure S4). The experimentally obtained compositions Zr_16.5(1)_Co_50.3(2)_B_33.2(3)_ and Hf_16.2(1)_Co_50.0(3)_B_33.8(3)_ (based on wavelength‐dispersive x‐ray spectroscopy, WDXS) are in close proximity to the nominal composition *TM*_16.7_Co_50.0_B_33.3_. Following the successful synthesis and manufacturing of the electrodes, the OER activities of ZrCo_3_B_2_ and HfCo_3_B_2_ were examined in the three‐electrode setup (Figures S5 and S6). Significantly lower OER overpotentials were detected for *TM*Co_3_B_2_ (ca. 270 mV for both) compared to the elemental Co electrode (ca. 320 mV, Figure 2a). Furthermore, comparable (in the case of ZrCo_3_B_2_) and almost twice higher (for HfCo_3_B_2_) current densities (compared to Co) were achieved initially at the highest measured potential (*E*_max_) of 1.95 V vs. RHE. At the same time, a noticeable (up to 30 mA cm^−2^ for both electrodes) offset current density at potentials close to the OER region points out the significant surface reconstruction before and at the initial stage of OER (inset of Figure 2a).

Considering the oxidative conditions of OER and material changes already observed in initial linear sweep voltammograms (LSVs), the reproducible and stable OER activity can be measured if the electrode material reaches the steady‐state. To reach the steady‐state as well as to monitor possible oxidation/reduction processes below the OER onset potential, cyclic voltammetry (CV) pre‐treatment applying potentials from 1.1 up to 1.6 V vs. RHE was carried out (Figure S7a). The CV cycles of ZrCo_3_B_2_, HfCo_3_B_2_ and reference Co show peaks around 1.4–1.45 V vs. RHE (Figure S7a,b), an indication of Co^3+^→Co^4+^ oxidation according to the literature data [39, 40]. Looking on the experimentally obtained CVs for Hf and Zr foils (Figure S7c,d) and considering the Pourbaix diagrams for these elements [41], no Zr and Hf oxidation is expected in this potential range. The measured high current density is a result of concurrent processes: OER, starting around 1.5 V vs. RHE, and oxidation of Co on the electrode surface (Figure S7a).

The current density values up to 0.7 A cm^−2^ for ZrCo_3_B_2_ and up to 1.1 A cm^−2^ for HfCo_3_B_2_ were obtained at *E*_max_ after CV pre‐treatment (Figure 2a). Such enhancement of the OER activity (compared to elemental Co) demonstrates a clear improvement of OER performance using the intermetallic compounds as precursors for OER‐active electrocatalysts. Apparently, the specific electronic and crystal structures of the investigated intermetallic compounds have a clear influence on the reactivity/functionality of the surface cobalt atoms under the applied reaction conditions. Along with OER activity, the electrocatalyst layer integrity as well as stability of OER activity over long periods of time are of tremendous importance. Therefore, chronopotentiometry (CP) at different conditions was performed. Short‐term CP (*st*‐CP) at benchmarking conditions (*j* = 10 mA cm^−2^, *t* = 2 h) clearly reveals the similar gap (∼ 100 mV) between measured potential values for elemental cobalt and intermetallic pre‐catalysts, anew justifying the improved OER electrocatalytic activity of studied materials compared to elemental Co (Figure 2b). Interestingly, the diminished offset current densities below onset OER potential after *st*‐CP can be considered as a quasi‐steady state of the electrocatalyst after CV pre‐treatment accompanied by surface reconstruction (Figure S8). The last but not least, the analysis of KOH solutions after *st*‐CP via inductively coupled plasma‐optical emission spectrometry (ICP‐OES) reveals only trace amount of Hf (0.2 mg L^−1^) in case of HfCo_3_B_2_ electrode (Table S2). The concentrations of other constituent elements in the electrolyte after OER experiments with the investigated electrodes are below the detection limits of ICP‐OES (< 0.05 mg L^−1^).

Considering the limitations of *st*‐CP, *TM*Co_3_B_2_ electrodes were exposed to the long‐term CP (*lt*‐CP) at elevated current densities (*j* = 50, 100, 200 mA cm^−2^) (Figure 2c,d). On one hand, both materials possess excellent OER activity up to 500 h of OER operation at elevated current densities, on the other hand, measured potentials reflect different responses of ZrCo_3_B_2_ and HfCo_3_B_2_ on applied conditions of *lt*‐CP. Contrary to HfCo_3_B_2_ slightly deactivating at lower current densities and showing stable OER activity at the highest applied current density of 200 mA cm^−2^, ZrCo_3_B_2_ reveals continuous slight (at 50 and 100 mA cm^−2^) and significant (at 200 mA cm^−2^) activation (Figure 2d). These results are also mirrored in the LSV data, recorded after each step of *lt*‐CP (Figure 2c). Almost constant potential values at an applied current density of 200 mA cm^−2^ with HfCo_3_B_2_ electrode (Figure 2d) highlight close to completed surface reconstruction. At the same time, continuous activation of the ZrCo_3_B_2_ anode (Figure 2d) points out its slower and non‐completed surface reconstruction compared to HfCo_3_B_2_. The ICP‐OES analysis of KOH aliquots after almost 500 h of *lt*‐CP (Table S2) also gave a hint about slow surface reconstruction and sustained material integrity, which was the biggest challenge for another Co‐containing material Mo_2_CoB_2_ exposed to deterioration of its OER activity due to the strong surface changes and continuous thickening of in situ formed layers of oxides [42]. No detectable amounts of Zr and B (< 0.05 mg L^−1^) were found in the spent KOH electrolyte after *lt*‐CP with ZrCo_3_B_2_ electrode (ICP‐OES data), whereas after the same experiment with HfCo_3_B_2_ the trace amounts of Hf (0.6 mg L^−1^) and B (0.2 mg L^−1^) were detected. These results are in line with the slower oxidation and in situ formation of Co active species in the upper most layers of the ZrCo_3_B_2_ electrode compared to HfCo_3_B_2_. Furthermore, the limited oxidation of Hf and B accompanied by dissolution allows the preservation of the electrode material integrity under harsh oxidative reaction conditions of OER. Also, it provides sufficient electron transfer in the system due to the presence of underlying HfCo_3_B_2_ bulk material with good electrical conductivity.

To shed light on the origin of the outstanding OER activity and its persistence over time, extensive insight into *TM*Co_3_B_2_ changes upon OER conditions via ex situ x‐ray photoelectron spectroscopy (XPS), SEM with energy‐dispersive x‐ray spectroscopy (EDXS), WDXS, and XRD was gained.

**FIGURE 3:**
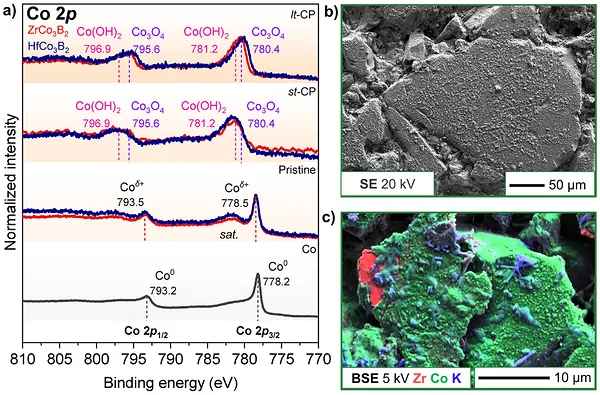
Pre‐ and post‐characterization of the ZrCo_3_B_2_ and HfCo_3_B_2_ electrodes: (a) XP Co 2*p* core level spectra in pristine states, after *st*‐ and *lt*‐CP measurements (elemental Co with Co 2*p*_3/2_ maximum at binding energy of 778.2 eV (in line with the literature value of 778.32(20) eV [49]) is added as reference); (b) SE image (20 kV) showing the surface morphology of the HfCo_3_B_2_ electrode after *lt*‐CP; (c) ZrCo_3_B_2_ electrode's BSE image (5 kV) with elemental mapping (after *lt*‐CP).

The different chemical interactions of the elements and as a result different electronic state of Co in HfCo_3_B_2_ compared to elemental Co are clear from the positive shift of the XPS Co 2*p* core levels toward higher binding energies (Co 2*p*_3/2_ at 778.5 eV) in HfCo_3_B_2_ compared to 778.2 eV for Co 2*p*_3/2_ in elemental Co (Figure 3a). After *st*‐CP only some part of Co*^δ^* ^+^ from HfCo_3_B_2_ electrode surface has been oxidized to the OER most active in situ‐existing cobalt (oxy)hydroxide CoOOH. After releasing the applied potentials and exposure to air, in situ‐formed upon anodic potentials CoOOH is reduced to Co_3_O_4_ (Co 2*p*_3/2_ at 780.4 eV) and Co(OH)_2_ (Co 2*p*_3/2_ at 781.2 eV), which are also in agreement with literature value of 780.4 eV [43] and 781.2 eV [44], respectively. From ex situ XP Co 2*p* core level spectra, Co(OH)_2_ is the dominant on the surface after *st*‐CP, which is most likely due to the incomplete surface oxidation of intermetallic HfCo_3_B_2_ during *st*‐CP and the passivation of residual Co*^δ^* ^+^ into Co(OH)_2_ under air in addition to that of formed during the reduction of CoOOH aforementioned. However, during the *lt*‐CP almost all surface Co*^δ^* ^+^ of HfCo_3_B_2_ pre‐catalyst was converted to the CoOOH, transforming to the Co_3_O_4_ after end of OER experiment. With the cobalt peak (Co 2*p*_3/2_) at 780.4 eV of Co_3_O_4_, the overwhelming in situ formation of state‐of‐art OER catalyst in alkaline media, Co_3_O_4_, is proved. These are very clear and solid results of the in situ modification of HfCo_3_B_2_ pre‐catalyst surface to the OER‐active species. The gradual depletion of Hf amount on the surface upon performed electrochemical experiments (based on XP Hf 4*d* and Hf 4*f* + K 3*p* core level spectra, Figure S9a,b) and absence of boron signals (from XP B 1*s* spectra, Figure S9c) support above‐described Co 2*p* XPS data and confirm predominant presence of in situ‐formed Co species on the top of OER‐exposed HfCo_3_B_2_. The XPS data collected from different spots of the HfCo_3_B_2_ electrode (Figure S10a) allow to prove the uniform distribution of in situ formed phases over the exposed surface (Figure S11).

The XP Co 2*p* core level spectrum of pristine intermetallic ZrCo_3_B_2_ contains clear peaks at binding energies of 778.5 and 793.5 eV, corresponding to Co 2*p*_3/2_ and Co 2*p*_1/2_ doublet lines, respectively, which are shifted by 0.3 eV to higher binding energies compared to elemental Co (Figure 3a). The shift of Co 2*p* core levels to higher binding energies is identical to that in HfCo_3_B_2_ compound. Moreover, the chemical nature of oxidized Co species on the surface of electrochemically exposed ZrCo_3_B_2_ is similar. The surface of ZrCo_3_B_2_ electrode after *st*‐CP is dominated by Co(OH)_2_ (Co 2*p*_3/2_ at 781.2 eV), whereas Co_3_O_4_ (Co 2*p*_3/2_ at 780.4 eV) forms as the surface layer after *lt*‐CP (Figure 3a). The detection of oxidized Zr in the form of ZrO_2_ (Zr 3*d*_5/2_ at 183.6 eV is close to literature value 183.3(1) eV [45]) and Zr(OH)_4_ as hydrolysis product of ZrO_2_ [46] (Zr 3*d*_5/2_ at 182.9 eV being identical to that of Ref. [47]), B(OH)_3_ with B 1*s* at 192.5 eV (192.8 eV [48]) and B_2_O_3_ with B 1*s* at 193.4 eV matching with 193.4 eV in Ref. [48] on the surface of ZrCo_3_B_2_ after *lt*‐CP (Figure S12) reveals that ZrCo_3_B_2_ oxidizes slower compared to the isostructural HfCo_3_B_2_ under applied reaction conditions. In addition, similar to HfCo_3_B_2_, a homogenously, uniformly distributed in situ‐formed phases has been observed (Figure S13).

Well‐preserved grains of HfCo_3_B_2_ precursor (Figure 3b) as well as overall integrity of the studied electrode (Figures S14 and S15a,b) point out the excellent bulk stability and integrity of the electrode. Having the optimal thickness of OER‐active phases at the surface in combination with the stable and conductive bulk material underneath is an ideal combination for outstanding long‐term OER performance. The EDXS analysis of HfCo_3_B_2_ electrode after *lt*‐CP using different acceleration voltages (probing different depth of the electrode) clearly reveals no Hf signal in the near‐surface region (acceleration voltage of 5 kV), whereas measurement deeper (at 15 kV) certainly shows the strong Hf Mαβ signal (Figure S15c). These results in combination with XPS and electrochemical data distinctly highlight the structural and chemical modifications of HfCo_3_B_2_ pre‐catalyst into an OER‐active Co‐rich phase under OER conditions. Furthermore, the WDXS line scan of Hf at 15 kV confirms only the significant Hf contribution at larger penetration depth, whereas such line scans of Co at 7 and 15 kV support its presence at bulk‐ and near‐surface regions (Figure S16, these results are in line with previously discussed XPS and EDXS data). Presented above analysis of the surface layers (via XPS), the near‐surface regions (using SEM) are accomplished by bulk‐sensitive characterization applying XRD in reflection mode. XRD data clearly revealed the unchanged positions of XRD peaks, corresponding to HfCo_3_B_2_.*r*, after electrochemical studies (Figure S17).

The ZrCo_3_B_2_ electrode after *lt*‐CP (Figure S18) was also subjected to the SEM analysis at acceleration voltages of 5 and 15 kV (Figure S19), illustrating similar features to those for HfCo_3_B_2_: no Zr signal at the near‐surface region and clear evidence in the bulk of studied electrode. On the other hand, Zr has been detected in the XPS measurement (Figures S12 and S13), which probes even much thinner layers compared to EDXS at 5 kV. Since reconstruction of ZrCo_3_B_2_ pre‐catalyst is much slower compared to that of HfCo_3_B_2_ and XPS probes a broader area, probably, the non‐changed parts of the pre‐catalyst (Zr‐rich part on Figure 3c (in *red*) and Figure S20 (in *white*)) or deposited zirconium oxide species (Figure S21) were detected in the XPS data. At the same time, the selected area for EDXS analysis (*red rectangle* on Figure S19a) represents the ZrCo_3_B_2_ grain, which undergo structural modification and, therefore, does not show Zr *L*α peak in the EDXS spectrum (5 kV). Upon ZrCo_3_B_2_ surface reconstruction, Zr*^δ^* ^+^ left the pre‐catalyst structure, allowing the formation of Co‐terminated surface, but afterward deposited ZrO_2_ grains were detected on the electrode surface (Figures S21 and S22). Zirconium line scans at near‐surface and at deeper penetration volume using WDXS analysis are in line with the quantitative EDXS analysis. Furthermore, the boron signals were obviously recognized from the WDXS analysis at 7 and 15 kV, confirming its presence in the near‐surface area (Figure S23). Even if the surface is enriched in Co, the bulk contains ZrCo_3_B_2_ (based on XRD data, Figure S24) as it is expected from its slow reconstruction upon OER.

**FIGURE 4:**
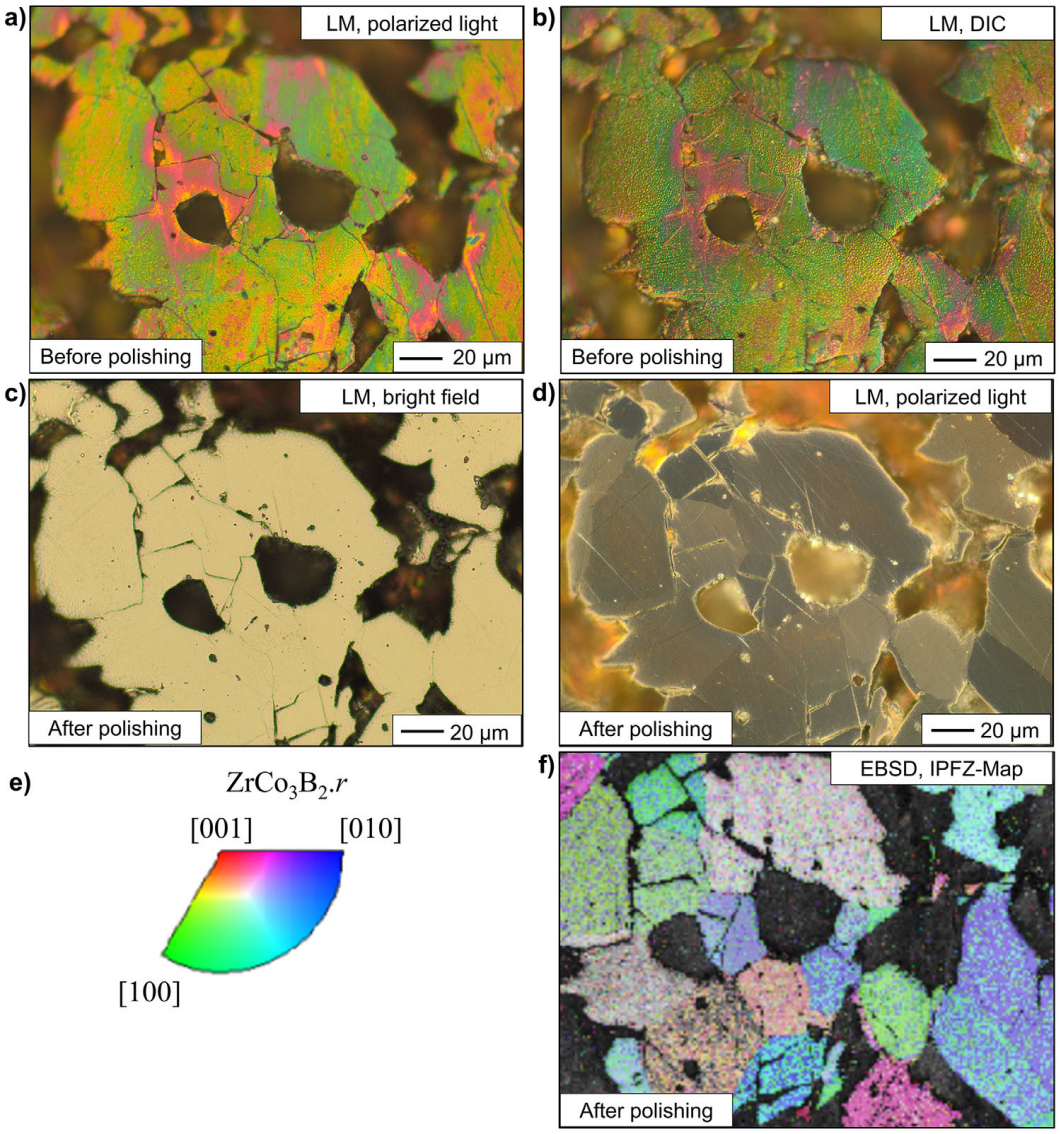
Light microscopy and EBSD characterization of ZrCo_3_B_2_ electrode after *lt*‐CP: the LM images in polarized light and differential interference contrast (DIC) mode before (a, b) and after polishing (c, d), and EBSD IPFZ (Inverse Pole Figure Z)‐map (*f*), supported by the color code for the orientation perpendicular to the sample surface (e).

The influence of preferred grain orientation (due to the possible anisotropy of bonding) onto chemical behavior/changes of electrode materials upon reaction conditions, correlation between the grain orientation and morphological changes on the surface during electrocatalysis was also investigated in a grain‐specific analysis of electrochemically treated area of *TM*Co_3_B_2_ performed via EBSD (Figure 4, Figures S25 and S26). While different colors of the selected grains on the light microscopy images with polarized light (Figure 4a, Figures S25a and S26a) give a hint about the varying thickness and/or nature of oxide layers on their surfaces, imaging with differential interference contrast (DIC) illustrated the morphological changes of the studied electrodes such as particle formation (Figure 4b, Figures S25b and S26b), being in line with the BSE images (Figure 3c and Figure S19b). Such combination of methods points out that all grains were exposed to similar surface reconstruction, there is no clear evidence of the influence of crystallographic orientation neither on the route nor on the products of oxidation upon OER.

## 3 Conclusion

The intermetallic precursors HfCo_3_B_2_ and ZrCo_3_B_2_ have demonstrated an outstanding OER activity and stability in terms of both electrocatalytic performance and material integrity. The surface reconstruction of both catalysts has enabled the in situ formation of electroactive cobalt oxide/(oxy)hydroxide species on the electrode surfaces, which then resulted in a significantly enhanced electrocatalytic performance with respect to elemental cobalt. With the lower onset overpotential (ca. 270 mV) and obtained huge current densities (0.7 A cm^−2^ for ZrCo_3_B_2_ and up to 1.1 A cm^−2^ for HfCo_3_B_2_) compared to the elemental Co, ZrCo_3_B_2_ and HfCo_3_B_2_ intermetallic compounds have shown their great potential toward alkaline OER. Last but not least, the formation of uniform, thin and dynamic OER‐active Co‐terminated layers in combination with the bulk stability and integrity of the *TM*Co_3_B_2_ (*TM* = Zr, Hf) electrode materials is a perfect strategy for obtaining high concentration of active centers on the surface leading to high current densities and the net electron transfer in the electrical circuit during the long‐term operation, bringing us one step forward in development of highly efficient OER electrocatalyst for large scale application of water electrolysis [50].

## 4 Methods

### 4.1 Synthesis

The intermetallic compounds *TM*Co_3_B_2_ (*TM* = Zr, Hf) were prepared from zirconium crystal bar (Haines & Maassen, Zr + Hf min. 99. 9%, Hf max. 0.5%), hafnium crystal bar (Haines & Maassen, Hf + Zr min. 99.9%, Zr max. 0.11%), cobalt slugs (Alfa Aesar, 99.95% metal basis), and boron crystalline powder (Alfa Aesar, 99.999% metal basis). These components were weighed in 1:3:2 atomic ratio (*TM*:Co:B), melted all together in an arc melter with a water‐cooled copper mold under partial pressure of argon. Sample homogeneity was ensured by melting of ingots with turn‐up a few times. Due to incongruent melting of ZrCo_3_B_2_ and HfCo_3_B_2_ [31], annealing of ingots was carried out at 1200°C for 7 days using the high‐temperature furnaces (HTM Reetz GmbH) positioned in the argon‐filled glove box ([O_2_] < 0.1 ppm, [H_2_O] < 0.1 ppm). The annealing of HfCo_3_B_2_ was extended to 21days to reach equilibrium state. Beforehand, the melted ingots were placed in ZrO_2_ crucible and sealed in Ta tube under argon.

### 4.2 Electrode Manufacturing

After successful synthesis of target bulk ZrCo_3_B_2_ and HfCo_3_B_2_ intermetallic compounds, the corresponding 8 mm dia. working electrodes were manufactured. The as‐synthesized materials were ground, well‐mixed, and placed in graphite die for spark‐plasma sintering (SPS, 515 ET Sinter Lab, Fuji Electronic Industrial Co. Ltd.). For efficient densification, the following SPS conditions were applied: *T*_max_ = 1000°C, dwelling at *T*_max_ for 15 min, and cooling down to room temperature just by switching off the SPS furnace. The sintered specimens were polished on LaboPol‐21 (Struers) polishing machine by using SiC paper (Struers, waterproof SiC #320, 46 µm grain size). To get a mirror‐lust electrode surface, the coarsely polished specimens were further polished on a satin‐woven natural silk plate (Struers, MD‐Dur, 250 mm dia.) with lubricant mixed diamond colloidal solutions (with particle sizes of 6, 3, and 1/4 µm, respectively) using the polishing machine (EcoMet250Pro, Buehler).

### 4.3 Characterization

Powder x‐ray diffraction (PXRD, Huber Imaging Plate Guinier Camera G670, Co Kα1 radiation, *λ* = 1.78896 Å) was employed for phase analysis of the synthesized samples in the as‐cast and annealed states. WinXPow software [51] was used for phase analysis via comparison of the experimental data with the calculated PXRD patterns. The lattice parameters of the target phases were refined from the PXRD patterns measured with internal standard LaB_6_ (*a* = 4.15692(1) Å) and applying WinCSD software package [52]. After *lt*‐CP, the electrodes were analyzed by x‐ray diffraction in reflection mode (STOE Stadi MP diffractometer, DECTRIS MYTHEN 1K detector, Cu Kα1 radiation, *λ* = 1.54060 Å).

The sample homogeneity and morphology of as‐synthesized and OER‐exposed *TM*Co_3_B_2_ (*TM* = Zr, Hf) materials were analyzed by light microscopy (LM, Zeiss Axioplan 2 light microscope, CCD camera, Olympus stream software [53]), scanning electron microscopy (SEM, JEOL JSM‐7800F microscope) with an energy‐dispersive x‐ray spectroscopy (EDXS, Quantax 400, X‐Flash 6|30 silicon drift detector, Bruker). The precise compositions of present phases were determined by the wavelength‐dispersive x‐ray spectroscopy (WDXS, CAMECA electron microprobe SX100 setup, W cathode, references: for Zr (ZrAsSe), Hf (elemental Hf with 1.6% Zr admixture), Co (elemental Co or CoSi), B (Ni_3_B)). To track the compositional changes of electrodes after OER experiments, WDXS was also performed in line scan mode. Bright field, polarized light, differential interference contract (DIC) using light microscope, backscattered electron (BSE) and secondary electron (SE) images, as well as elemental mappings applying SEM were recorded to observe the changes in morphology and composition of the *TM*Co_3_B_2_ electrodes after OER experiments. The BSE and SE images were processed using Esprit 2.3 software [54]. Electron backscatter diffraction (EBSD) system at SEM (JSM‐7800F with Bruker CrystAlign 400 EBSD electron microscope analyzer) was used to shed light onto the co‐existing crystal structure types (*TM*Co_3_B_2_.*r* or *TM*Co_3_B_2_.*h*) in the *TM*Co_3_B_2_ samples as well as to obtain information about crystallographic orientations of the grains.

To determine the electronic state of constituent elements in the pristine *TM*Co_3_B_2_ materials and to track their changes during OER experiments, x‐ray photoelectron spectroscopy (XPS) was applied. The XPS spectrometer is equipped with a twin crystal monochromatized Al Kα source (*hv* = 1486.6 eV) and an electron energy analyzer (Scienta R3000, in normal emission geometry). All measurements were conducted at room temperature, and the maintenance of the pressure in the XPS chamber within 10^−10^ mbar range was ensured. The overall energy resolution was around 0.4 eV, and the calibration of the Fermi level was with respect to a polycrystalline Ag reference.

After each OER experiment, two aliquots of the exploited KOH electrolyte were taken, and the concentrations of the dissolved constituent elements (Zr, Hf, Co and B) were analyzed vs. blank KOH by the inductively coupled plasma‐optical emission spectrometry (ICP‐OES, Agilent 5100 SVDV spectrometer).

### 4.4 Electrochemical Experiments

The OER experiments were performed in three‐electrode PEEK cell (Figure S5) filled with 1 M KOH (Thermo Fisher Scientific) and purged with Argon 5.0 flow for at least 20 min. The as‐synthesized *TM*Co_3_B_2_ (*TM* = Zr, Hf) as working electrode, the reference Hg/HgO electrode (PINE Research, 9.5 mm tip, stored in 1 m KOH), and Pt wire as counter electrode (PINE Research, 4.9 cm^2^ surface area of coil)were assembled together for the OER experiments. The polishing of the electrode surfaces (as described above) was carried out prior to each measurement. The reference Hg/HgO electrode was calibrated in 1 m KOH electrolyte against H^+^/H_2_ reference hydrogen electrode (RHE, Gaskatel, HydroFlex) on the daily basis. SP‐300 potentiostat (Bio‐Logic Science Instruments) and EC‐Lab software [55] were employed to carry out OER experiments and record the EC data. Data was processed using Origin(Pro) [56]. The OER activity measurements were initiated with open circuit voltage (OCV, ∼ 10 min), continued with linear sweep voltammetry (LSV, *E*_min_ = 0.6 V vs. RHE, *E*_max_ = 2.0 V vs. RHE, scan rate of 5 mV s^−1^) for evaluation of initial OER activity, followed by cyclic voltammetry electrode pre‐treatment (CV, *E*_min_ = 1.0 V vs. RHE, *E*_max_ = 1.5 V vs. RHE, 50 cycles, scan rate of 50 mV s^−1^), and finalized with another LSV at the same conditions (to assess the impact of electrode pre‐treatment onto the OER activity of studied electrodes). The stability of OER activity was estimated in two different modes: (i) standard chronopotentiometry (*st*‐CP, OCV for 1 min, followed by CP at current density of 10 mA cm^−2^ for 2 h), and ended with OER activity measurement by LSV (see above); and (ii) long‐term CP (*lt*‐CP, started with OCV for 10 min, followed by stepwise increase of current density from 50 to 100 mA cm^−2^, and, finally, to 200 mA cm^−2^ during CP for extended periods of time with final check of OER activity by LSV (Figure S6).

### 4.5 Calculations

Calculations of electronic structure and analysis of chemical bonding were performed with the all‐electron, full‐potential local orbital method (FPLO [57], local density approximation and the Perdew‒Wang parametrization [58], spin‐polarized scalar relativistic calculation, standard basis set, 12 × 12 × 12 k points). The experimental atomic coordinates obtained from crystal structure refinement of ZrCo_3_B_2_.*r* [59] were used for calculations. The coordinates for HfCo_3_B_2_.*r* from the structure determination [33] were optimized keeping the experimental lattice parameters constant, using the FPLO procedures. Spin‐polarized calculations were performed. For the study of chemical bonding, the electron density (ED) and the electron localizability indicator (ELI‐D) were calculated with a specialized module implemented in the FPLO program package [60]. The topology of ED and ELI‐D was analyzed with the program DGrid [61]. The ED was integrated within atomic basins, i.e., spatial regions confined by zero‐flux surfaces in the gradient field of ED and ELI‐D, respectively. This technique represents the procedure proposed in the Quantum Theory of Atoms in Molecules (QTAIM [35]) and provides effective electron populations for the QTAIM atoms and ELI‐D bond basins. Further information about the bonding between atoms is obtained from combined analysis of ED and ELI‐D (electron localizability approach [34]).

## Author Contributions

**Iryna Antonyshyn**: project administration, supervision, writing – original draft, investigation, data curation, visualization, validation, methodology. **Yuri Grin**: conceptualization, writing – original draft, supervision, visualization, investigation, project administration, resources, validation, funding acquisition, methodology. **Fatma Aras**: investigation, data curation, writing – original draft, visualization, methodology, formal analysis, funding acquisition. **Simone G. Altendorf**: investigation, visualization, writing – review and editing, formal analysis. **Ulrich Burkhardt**: investigation, writing – review and editing, visualization, formal analysis, validation.

## Conflicts of Interest

F.A., U. B., I. A. and Y. G. are the inventors of patent (WO 2025/017013 A1), S.G. A. declares no competing interests. There is no conflict of interest.

## Supporting information




**Supporting File**: advs77373‐sup‐0001‐SuppMat.pdf.

## Data Availability

The data that support the findings of this study are available on request from the corresponding authors.
