# Supplementary material for: ZrCo3B2 and HfCo3B2 Electrodes Go on Stage for Oxygen Evolution Reaction
Source: Adv Sci (Weinh). 2026 Sep 16:e77373. Online ahead of print. doi: 10.1002/advs.77373 (PMC13580386; doi:10.1002/advs.77373)
Supplement: Supplementary file 1 [file ADVS-9999-e77373-s001.pdf]

Supporting Information

$ZrCo_3B_2$ and $HfCo_3B_2$ Electrodes Go on Stage for Oxygen Evolution Reaction

Fatma Aras[1,*], *Ulrich Burkhardt*[1], *Simone G. Altendorf*[1], *Yuri Grin*[1], *and Iryna Antonyshyn*[1,2,*]

[1]Max-Planck-Institut für Chemische Physik fester Stoffe, Dresden, Germany

[2]Fritz-Haber-Institut der Max-Planck-Gesellschaft, Berlin, Germany

Corresponding Authors*: Fatma Aras (Fatma.Aras@cpfs.mpg.de) | Iryna Antonyshyn (Iryna.Antonyshyn@cpfs.mpg.de)

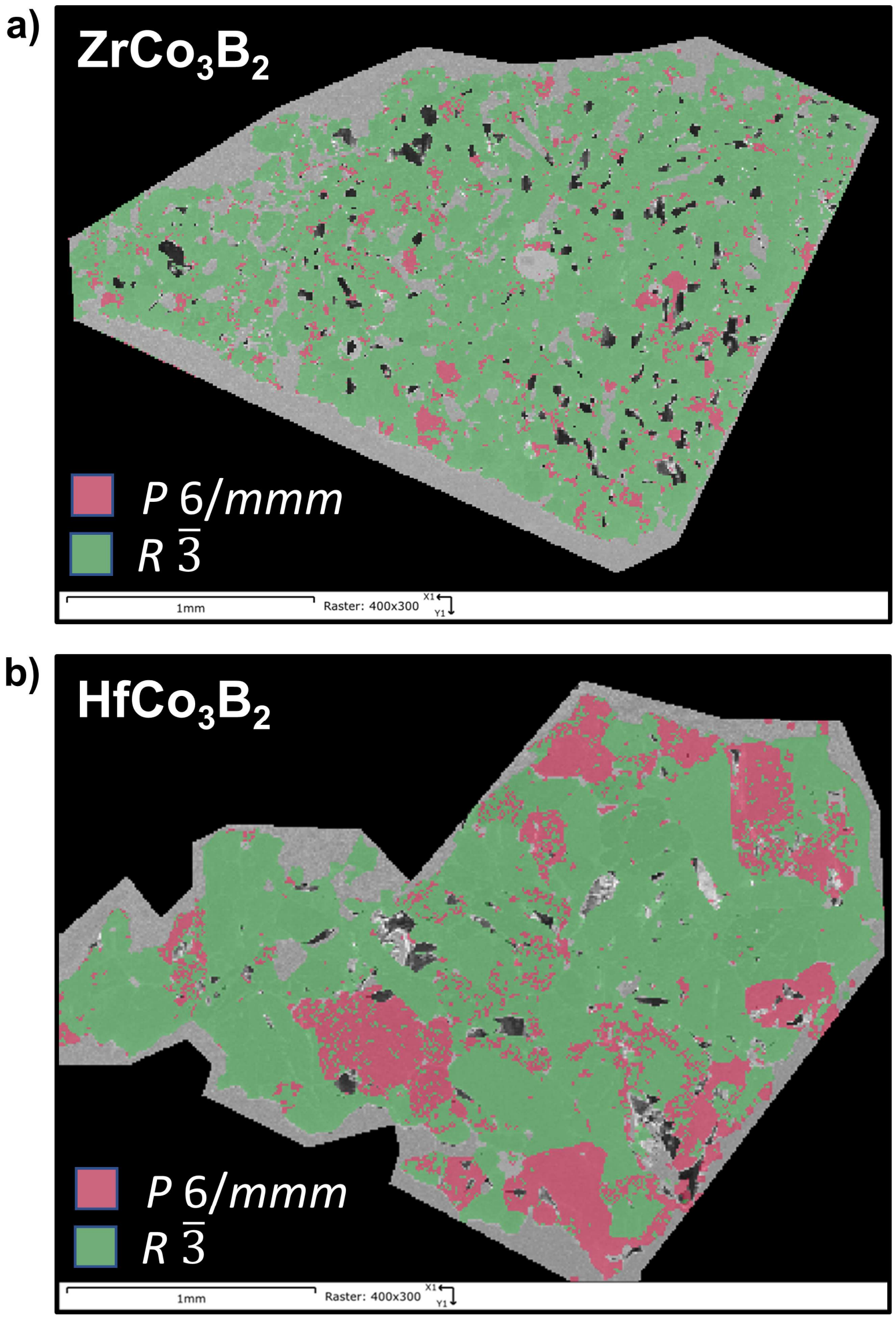

Figure S1. EBSD analysis of the embedded pristine samples $ZrCo_3B_2$ (*a*) and $HfCo_3B_2$ (*b*). The grains possessing the hexagonal structure (type $CeCo_3B_2$, space group *P*6/*mmm*) are in *pink* and those with the rhombohedral structure ($ZrCo_3B_2$ type, space group $R\bar{3}$) – in *green*. The non-resolved areas are in *grey*.

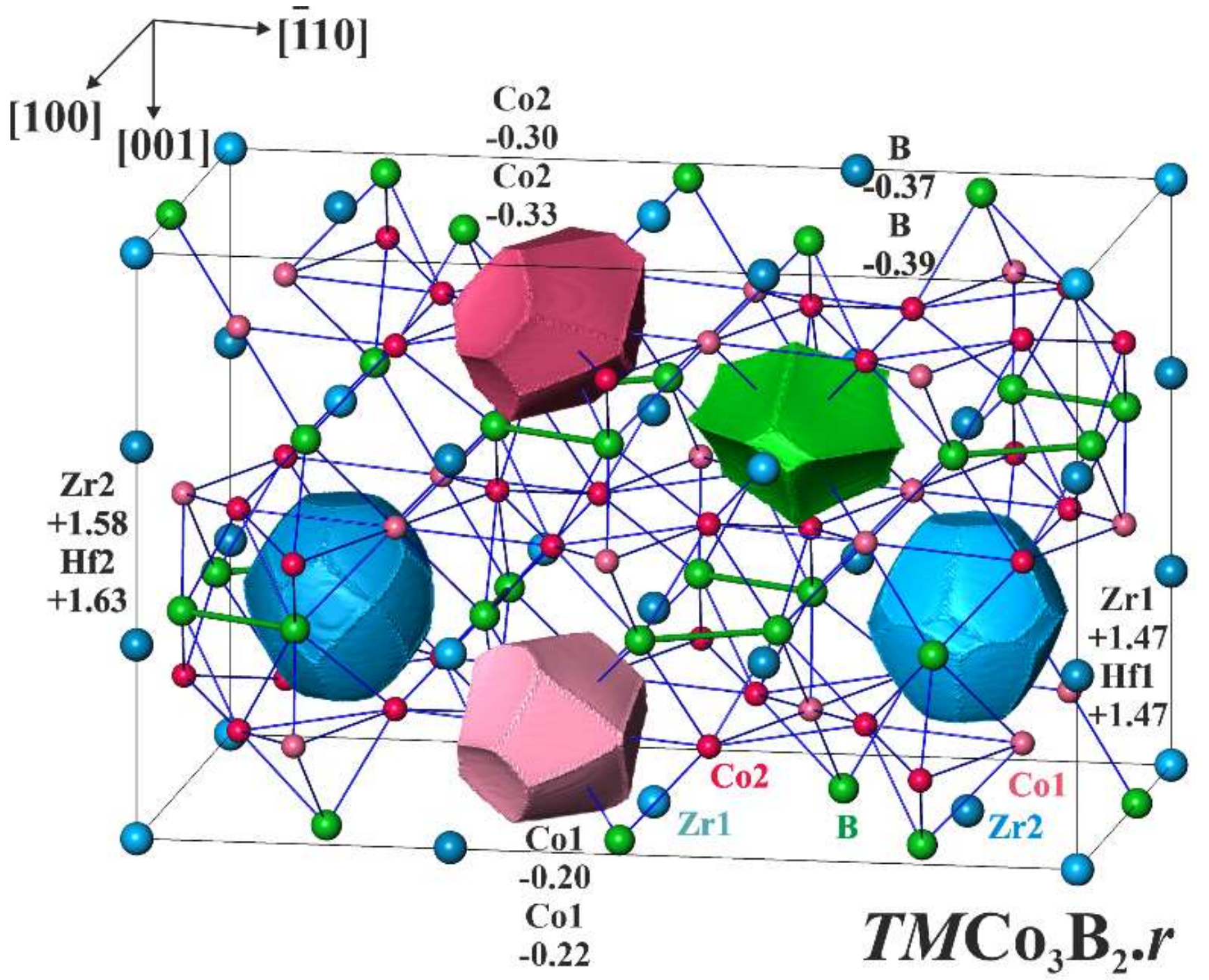

Figure S2. Shapes and effective charges of QTAIM atoms in *TM*Co_3B_2.*r* (*TM* = Zr, Hf) compounds. The values in *upper line* correspond to $ZrCo_3B_2$.*r*, and in *lower line* – to $HfCo_3B_2$.*r* compound.

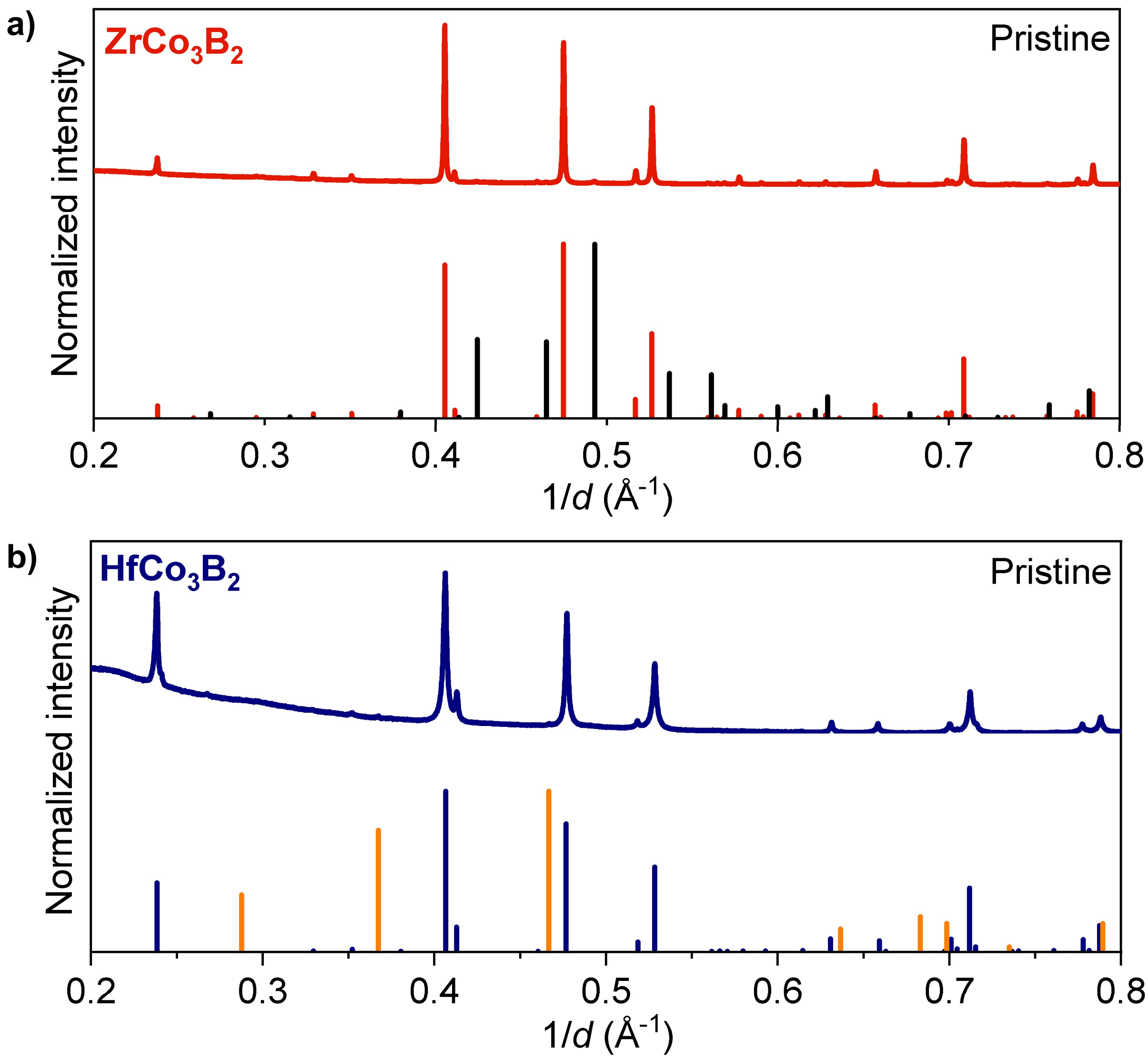

Figure S3. Experimental PXRD patterns of pristine $ZrCo_3B_2$ (*a*, *red*) and $HfCo_3B_2$ (*b*, *navy*). The lines of corresponding colors below represent the peak positions in calculated patterns for *TM*Co_3B_2.*r*. The traces of $Zr_2Co_{21}B_6$ and HfB_2 (on the experimental PXRD patterns of $ZrCo_3B_2$ and $HfCo_3B_2$, respectively) are obvious only based on the most intense reflections in the calculated patterns (*black and orange lines below, respectively*). PXRD measurements were performed in transmission mode (Co $K\alpha_1$ radiation, λ = 1.78896 Å).

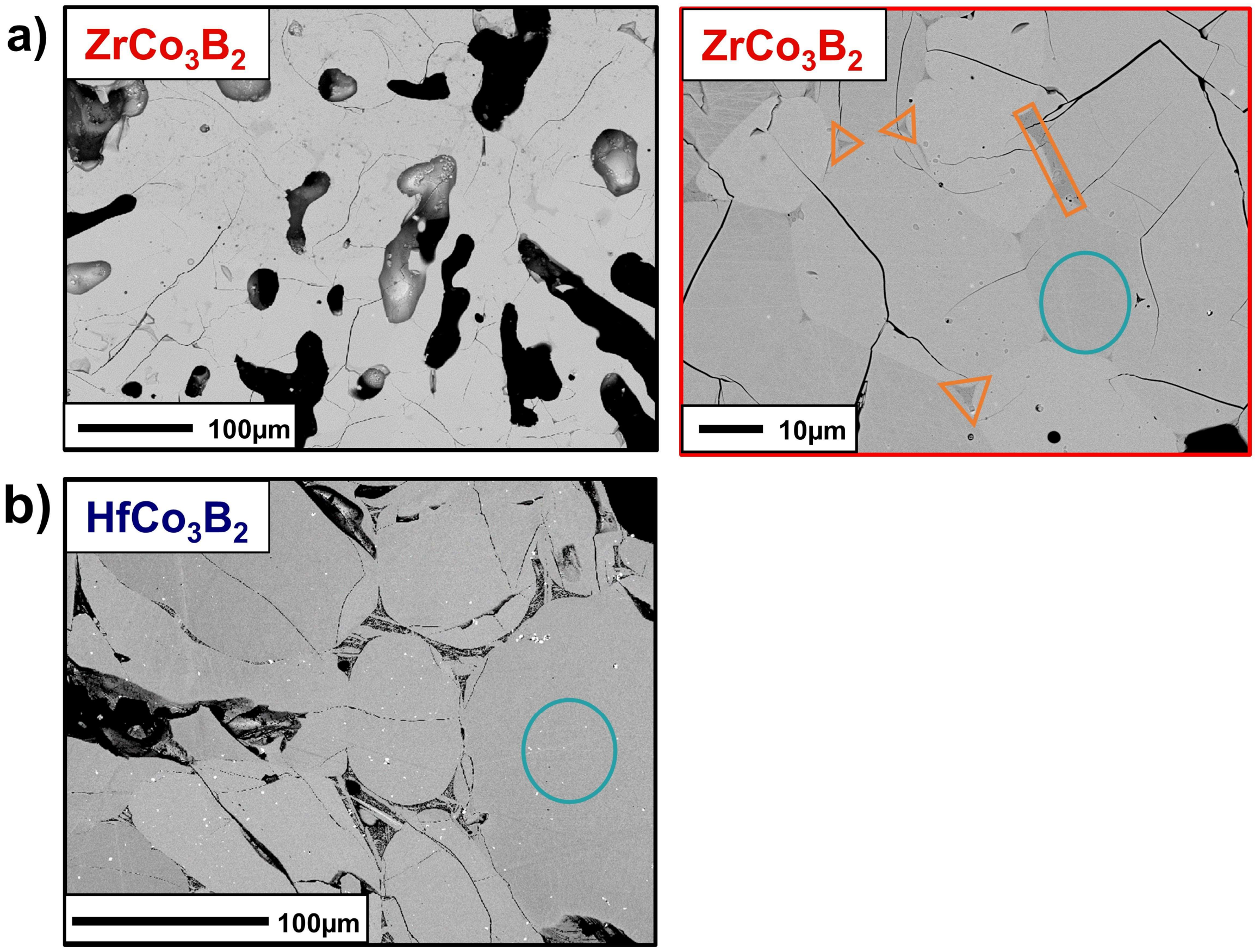

Figure S4. BSE images of pristine $ZrCo_3B_2$ (*a*) and $HfCo_3B_2$ (*b*). *Turquoise circles* represent the main *TM*Co_3B_2 phases and *orange rectangle/triangles* show $Zr_2Co_{21}B_6$ secondary phase in the enlarged area of $ZrCo_3B_2$ specimen (*red* frame).

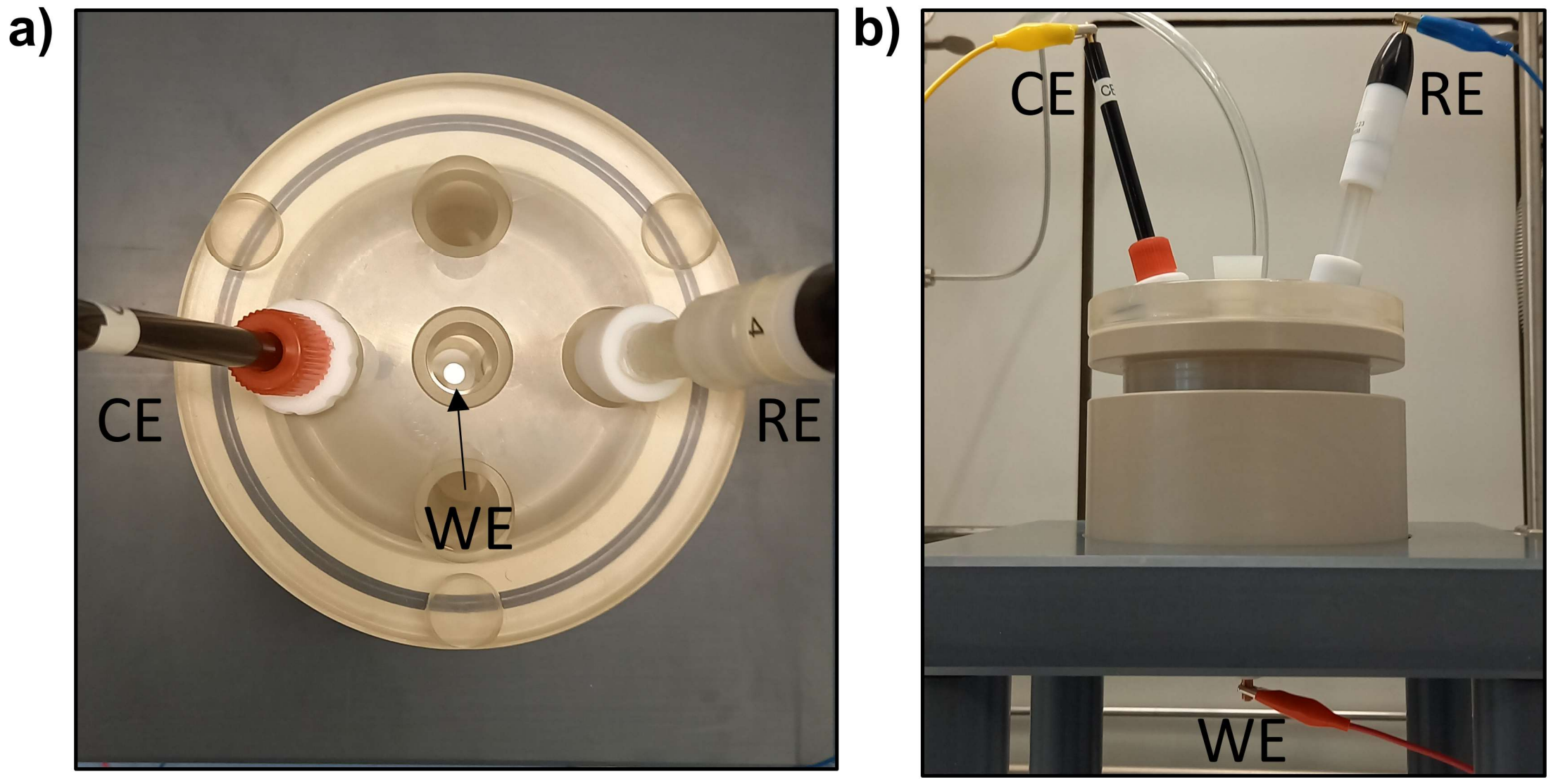

Figure S5. Top (*a*) and front (*b*) views of the three-electrode setup used for the electrochemical measurements. The following abbreviations are used: CE - counter electrode, RE - reference electrode and WE - working electrode.

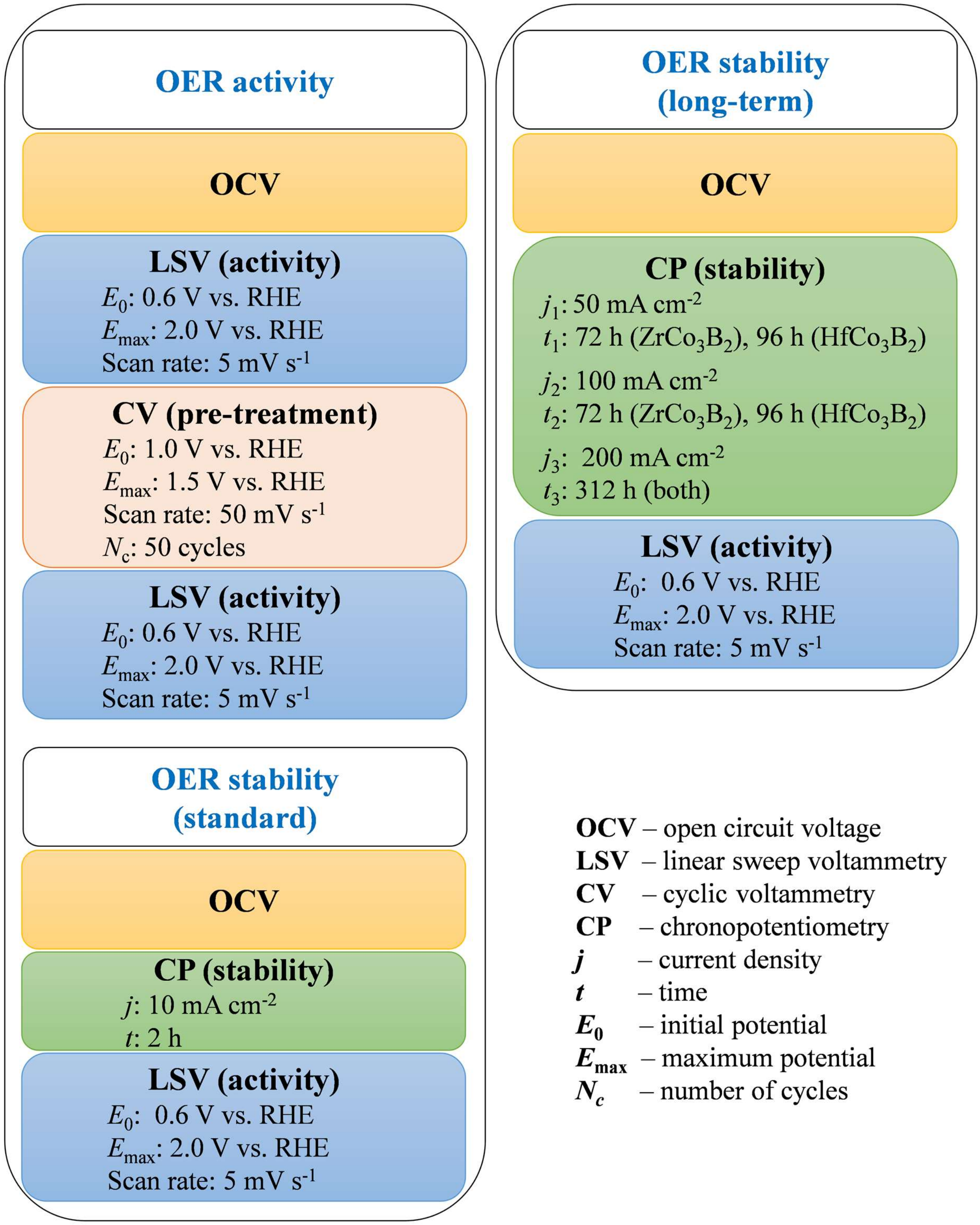

Figure S6. The sequence of applied electrochemical techniques for OER activity and stability assessment of the studied $TMCo_3B_2$ (TM = Zr, Hf) electrodes. The techniques applied subsequently are framed together.

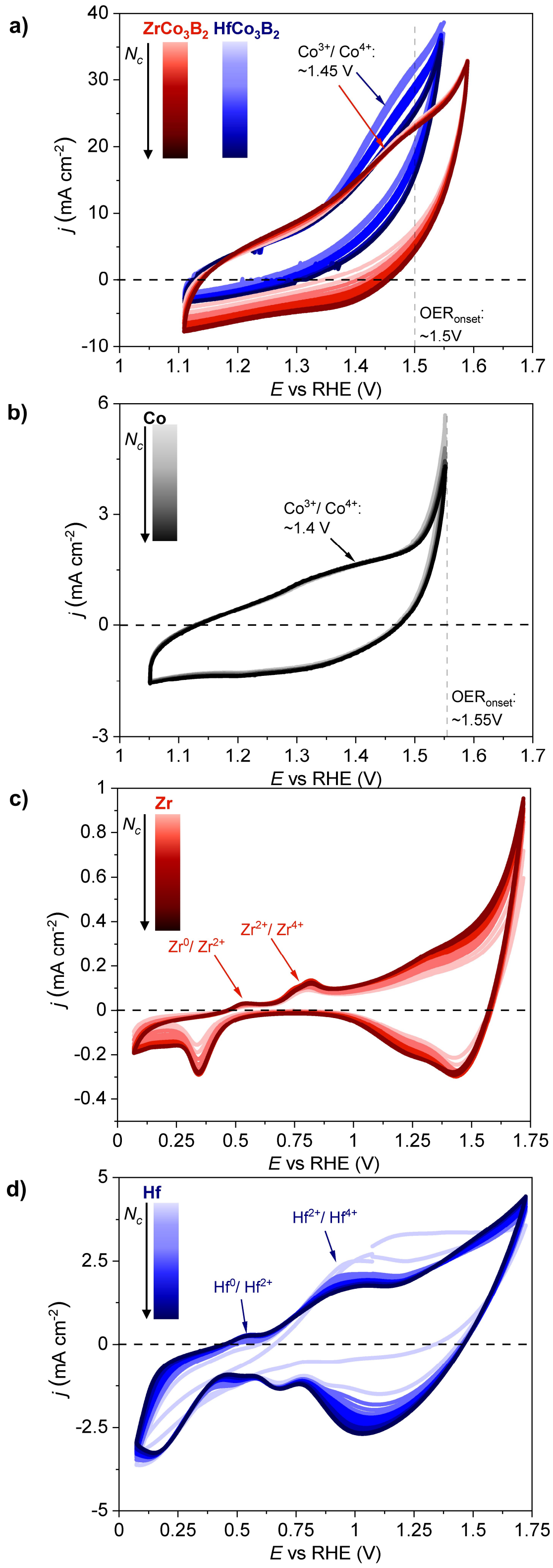

Figure S7. Cyclic voltammetry pre-treatment of $HfCo_3B_2$ (*a*, *navy*), $ZrCo_3B_2$ (*a*, *red*) and Co (*b*, *black*). *Horizontal dashed line* guides the eye between the anodic and cathodic regions, *arrows* show the oxidation peaks with the increasing number of CV cycles. The cyclic voltammograms of Zr (*c*) and Hf (*d*) foils were measured as references in the broader potential range (from 0.07 to 1.72 V vs. RHE). The increase of obtained current density of Zr (between 1.5-1.72 V vs. RHE) is considered due to the dissolution of zirconium oxide species in the alkaline medium [1].

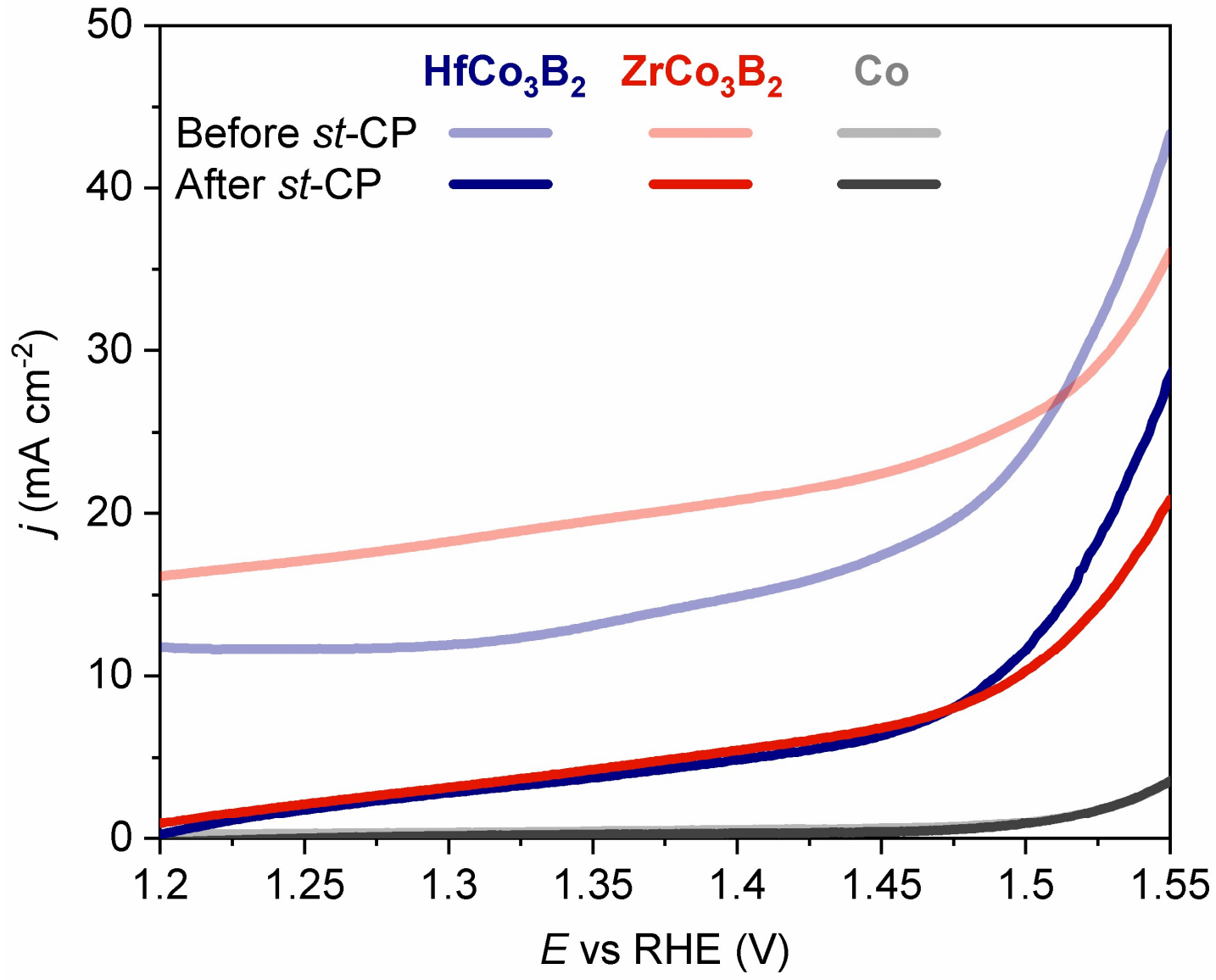

Figure S8. LSVs with $HfCo_3B_2$ (*navy*), $ZrCo_3B_2$ (*red*) and Co (*black*) electrodes before (*light colors*) and after (*dark colors*) *st*-CP (j = 10 mA cm^{-2}, t = 2 h).

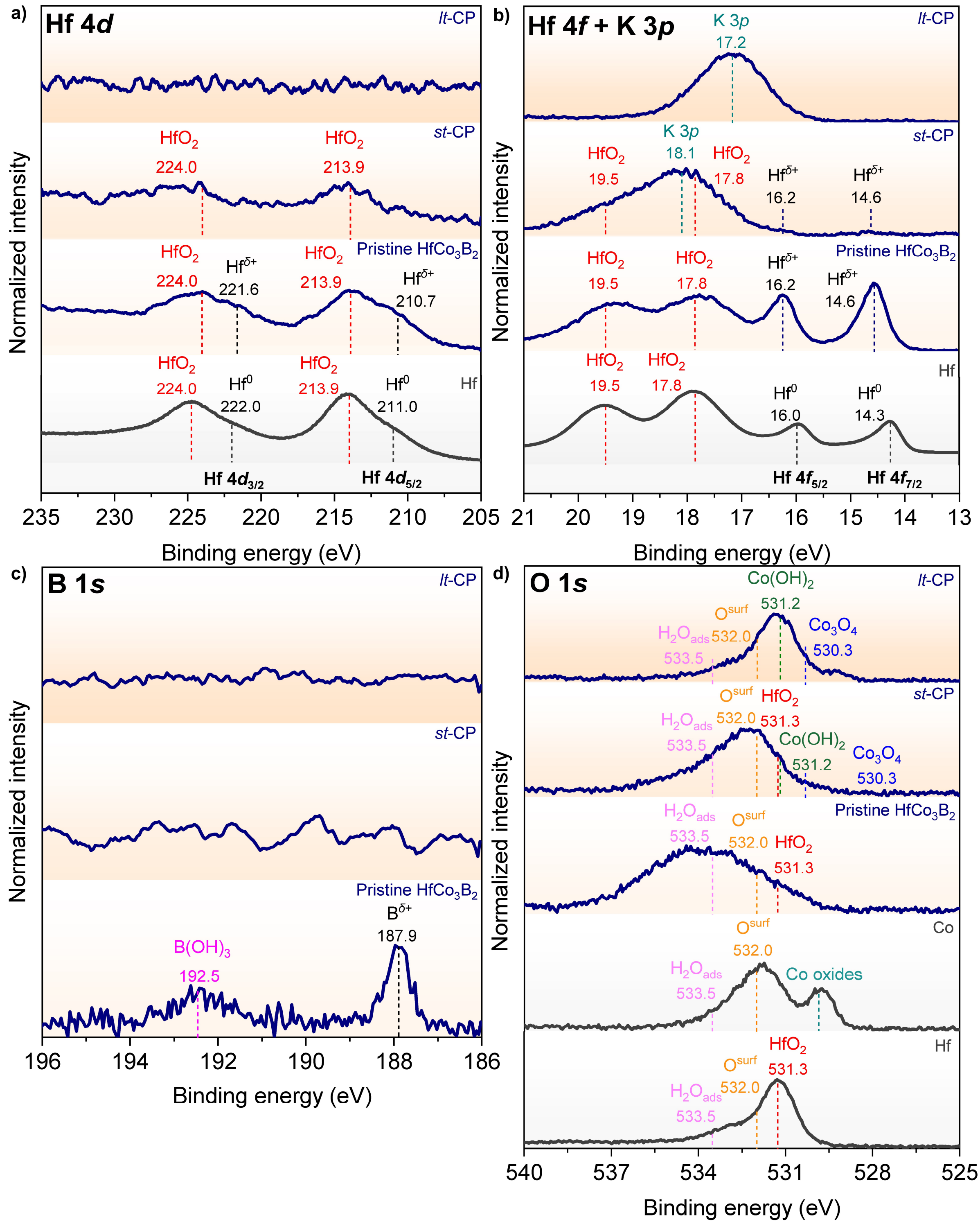

Figure S9. XP Hf 4*d* (*a*), Hf 4*f* + K 3*p* (*b*), B 1*s* (*c*) and O 1*s* (*d*) core level spectra of $HfCo_3B_2$ in pristine form and after different CP experiments (results for the electrode after *lt*-CP are presented for position 2 from **Figure S10a**). The XP Hf 4*d*, Hf 4*f* + K 3*p* and O 1*s* core level spectra for reference Co and Hf are added for better comparison. The labelled peak positions for different compounds are taken from the following references: Hf^0 [2,3], HfO_2 [3,4], $B(OH)_3$ [O 1*s* for hydroxides: 531-532 eV (considered for $B(OH)_3$)] [2, 5], CoO [6], $Co(OH)_2$ [7], Co_3O_4 [8], O^{surf} (non-stoichiometric surface region oxygen) [9], H_2O_{ads} (adsorbed water either from air or reaction medium) [8].

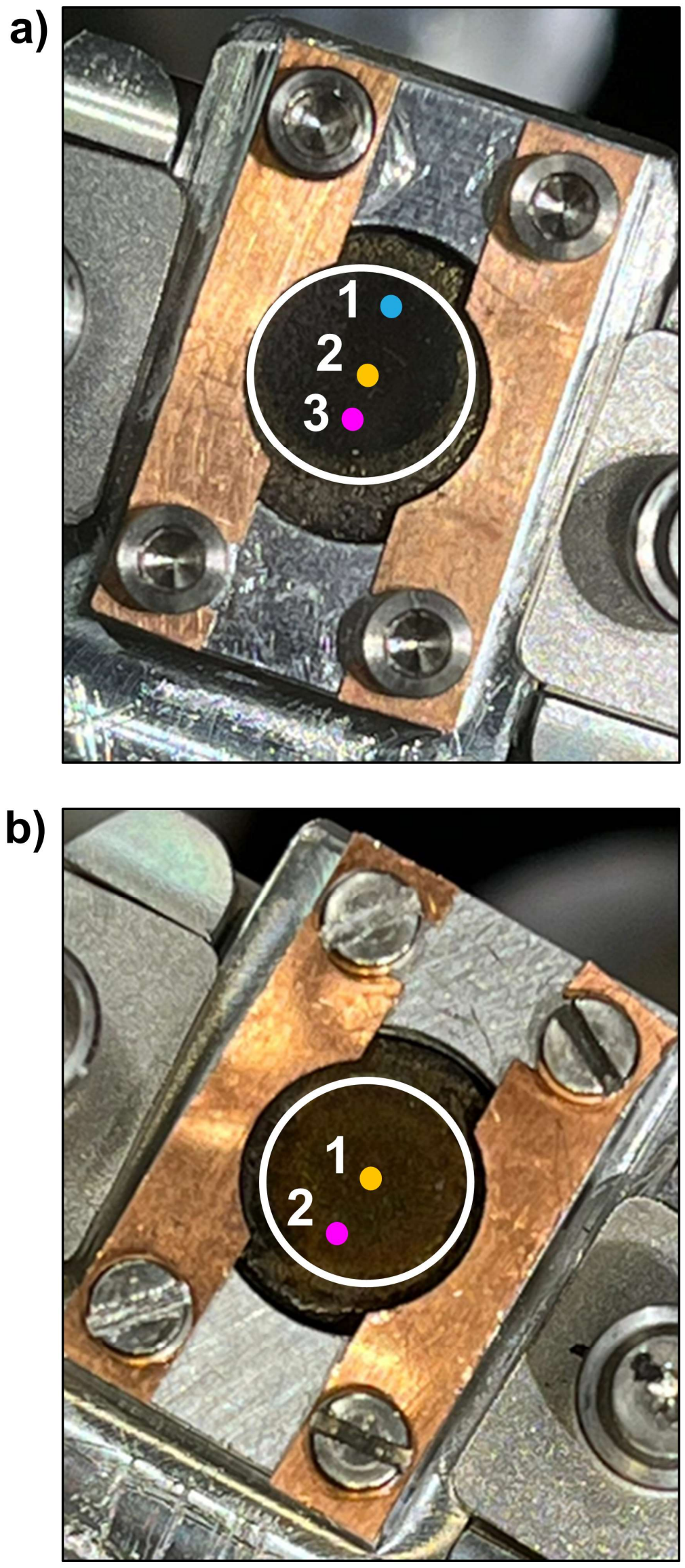

Figure S10. XPS-measured spots on $HfCo_3B_2$ (*a*) and $ZrCo_3B_2$ (*b*) electrodes after *lt*-CP.

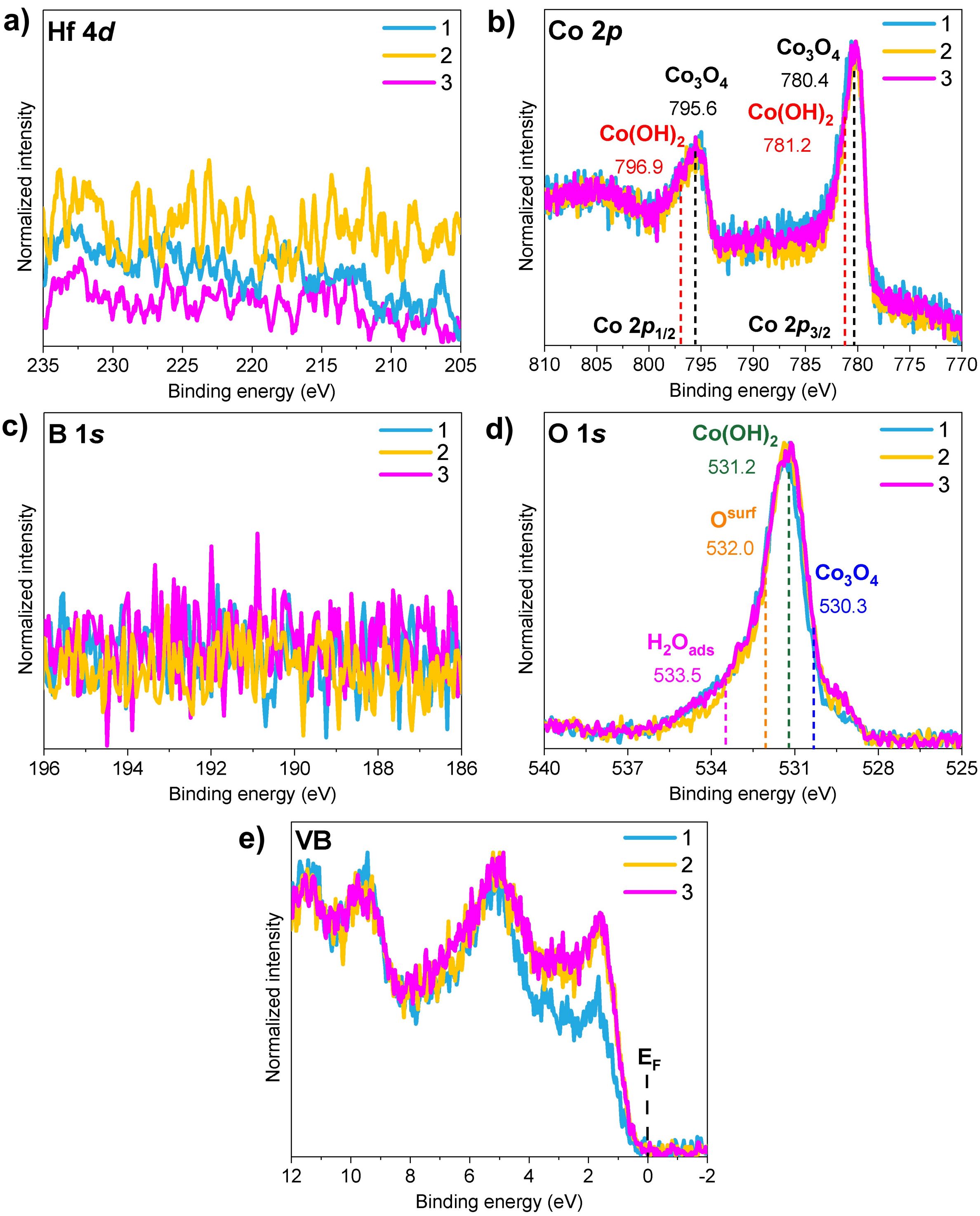

Figure S11. XP Hf 4*d* (*a*), Co 2*p* (*b*), B 1*s* (*c*), O 1*s* (*d*) core level and VB (*e*) spectra collected from different spots of $HfCo_3B_2$ electrode (see **Figure S10a)** after *lt*-CP.

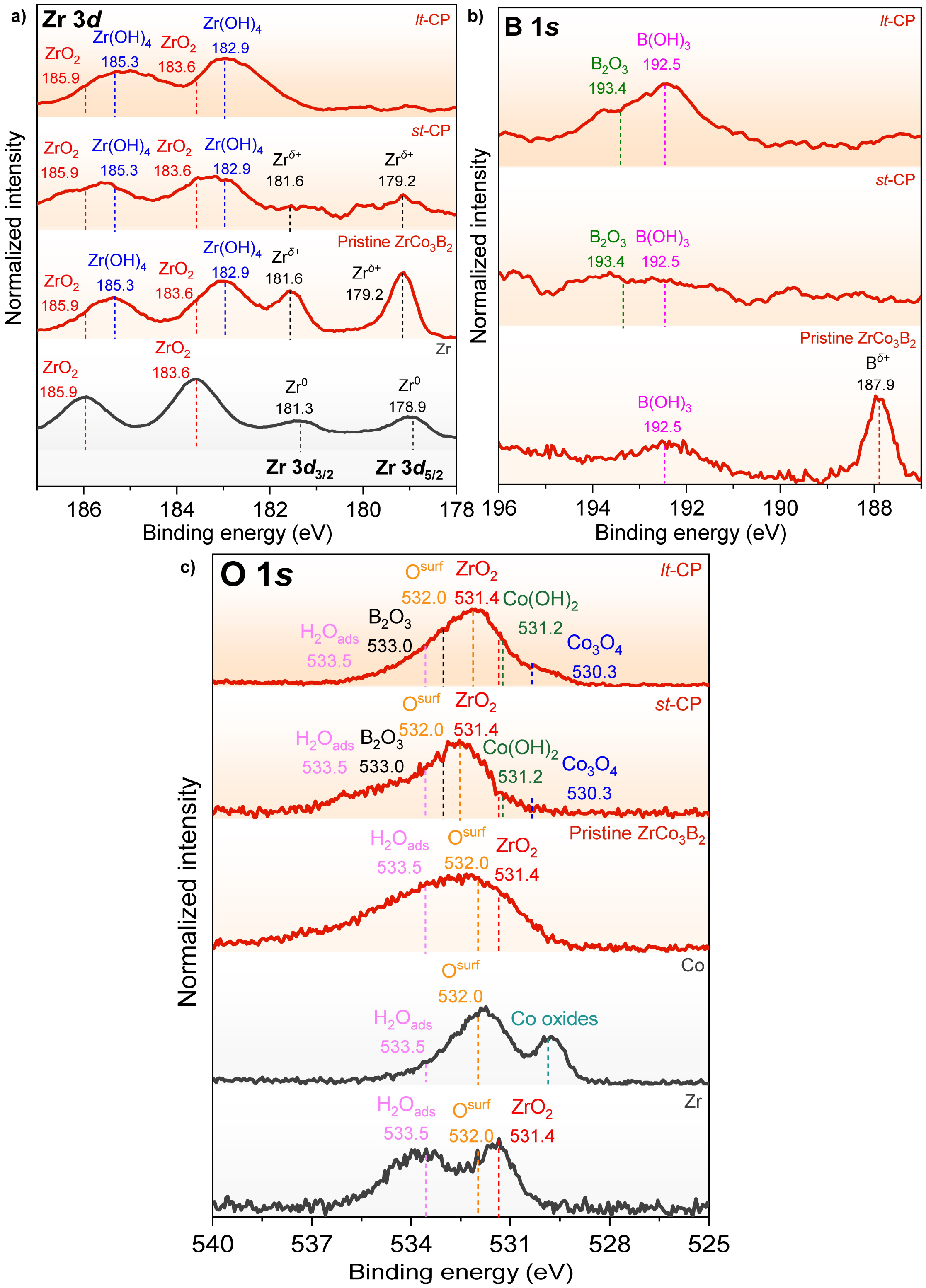

Figure S12. XP Zr 3*d* (*a*), B 1*s* (*b*) and O 1*s* (*c*) core level spectra of $ZrCo_3B_2$ in pristine state and after different CP experiments (data after *lt*-CP are presented for position 2, see **Figure S10b**). The XP Zr 3*d* and O 1*s* core level spectra for reference Co and Zr are added for better comparison. The peak positions for different compounds are assigned based on the following references: Zr^0 [2], ZrO_2 [10], $Zr(OH)_4$ [11], $B(OH)_3$ and B_2O_3 [5] [O 1*s* for hydroxides: 531-532 eV (considered for $Zr(OH)_4$ and $B(OH)_3$)] [2,5], CoO [6], $Co(OH)_2$ [7], Co_3O_4 [8], O^{surf} (non-stoichiometric surface region oxygen) [9], H_2O_{ads} (adsorbed water wither from air or reaction medium) [8].

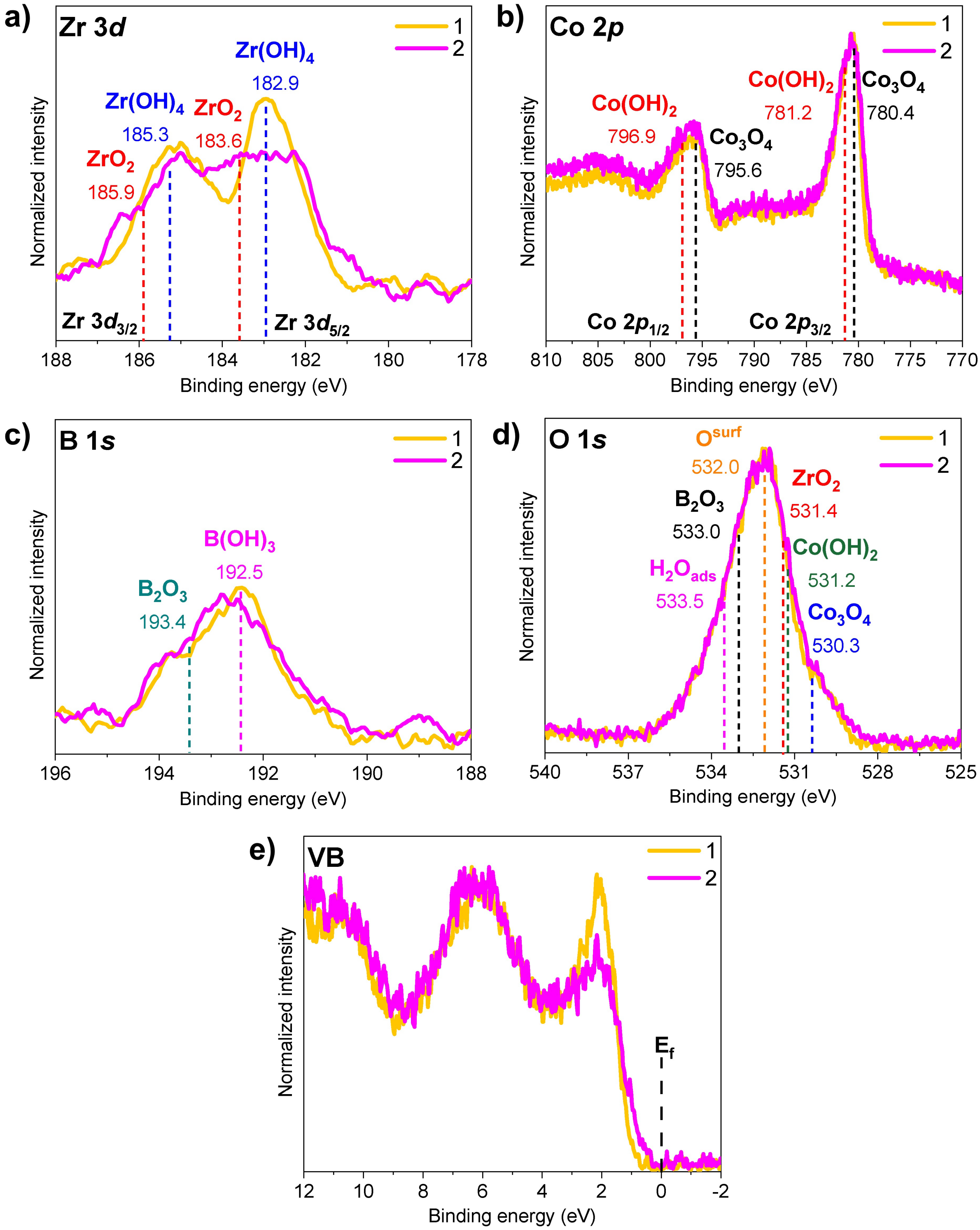

Figure S13. XP Zr 3*d* (*a*), Co 2*p* (*b*), B 1*s* (*c*), O 1*s* (*d*) core level and VB (*e*) spectra of $ZrCo_3B_2$ electrode after *lt*-CP measured at different spots (see **Figure S10b**).

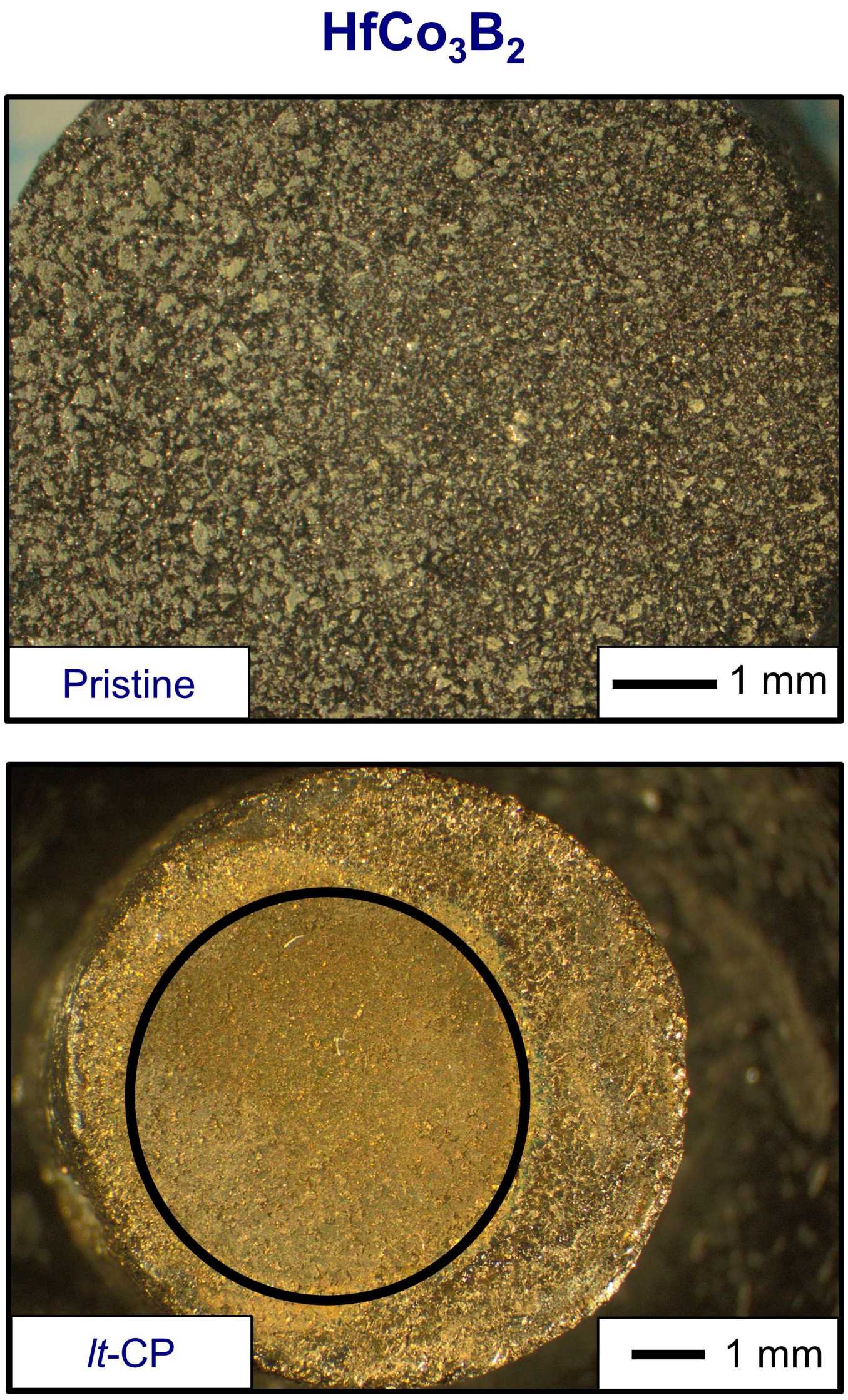

Figure S14. Light microscopy images (bright field) of $HfCo_3B_2$ electrode before and after *lt*-CP.

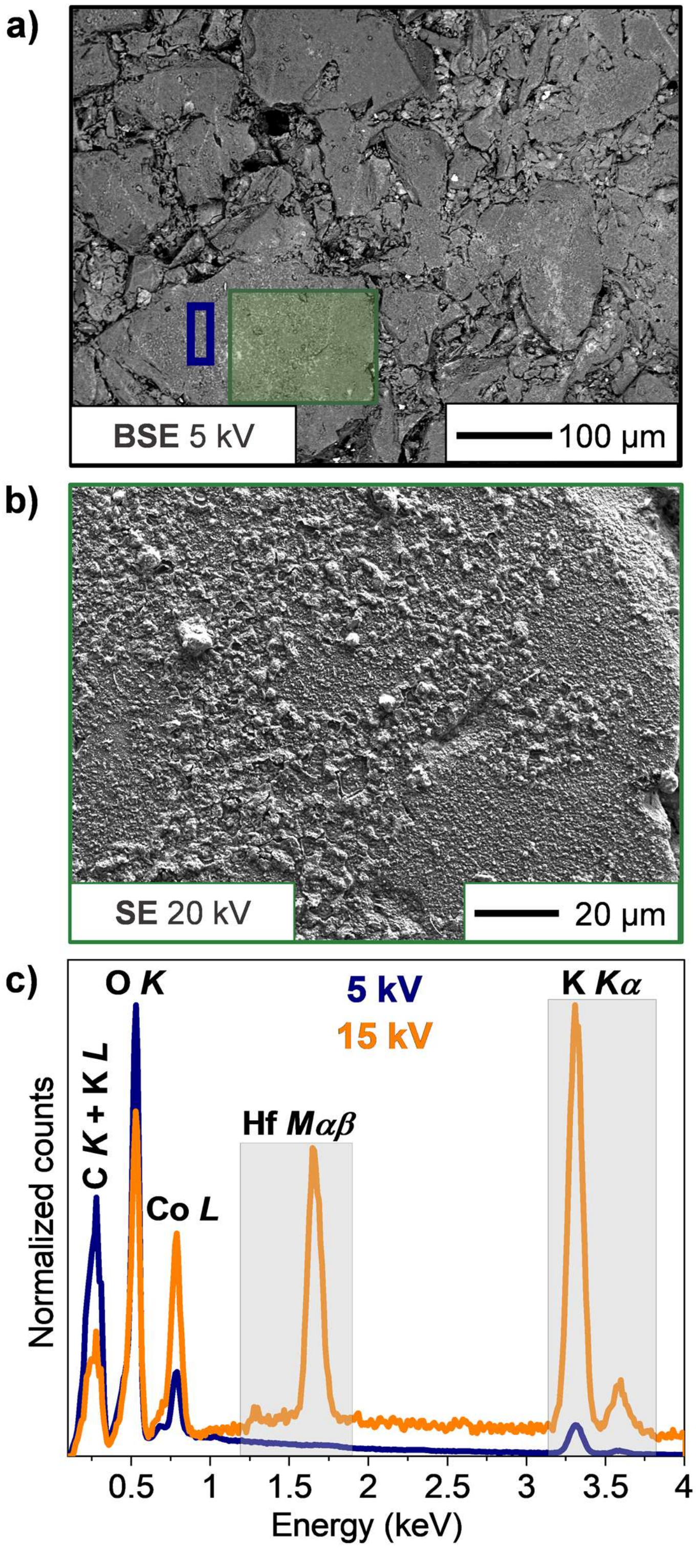

Figure S15. SEM characterization of $HfCo_3B_2$ electrode after *lt*-CP: (*a*) BSE image (5 kV) showing the area where EDXS spectra were measured (*blue rectangle*); (*b*) SE image (20 kV) showing the surface morphology from the top of electrode (*green rectangle* represents the enlarged area in the following image (from *top* to *bottom*)); (*c*) measured EDXS spectra with different acceleration voltages (5 and 15 kV).

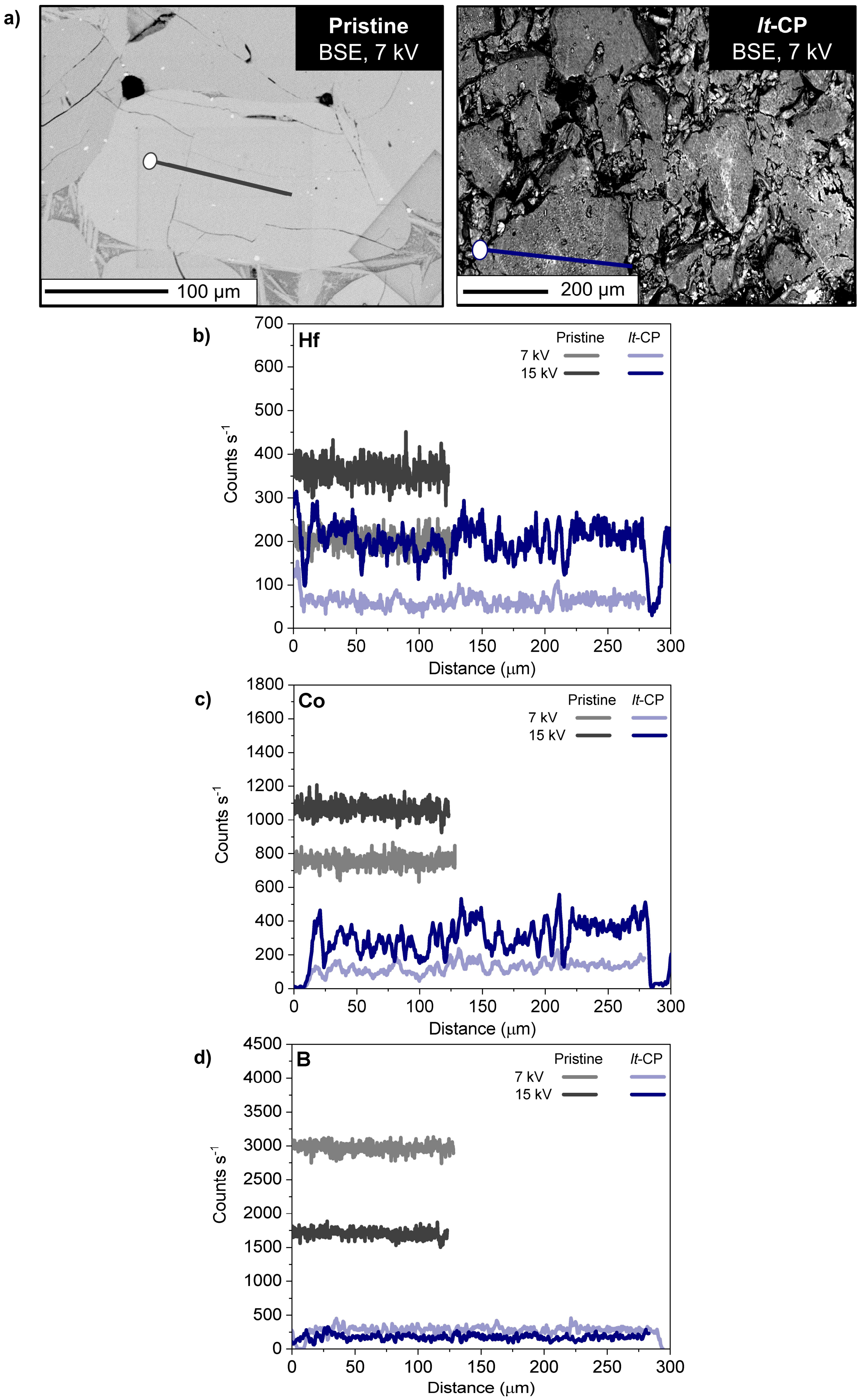

Figure S16. BSE images (*a*) and WDXS line scans (*b*-*d*) of $HfCo_3B_2$ electrode before (*grey*) and after (*navy*) *lt*-CP, measured at different acceleration voltages (7 and 15 kV).

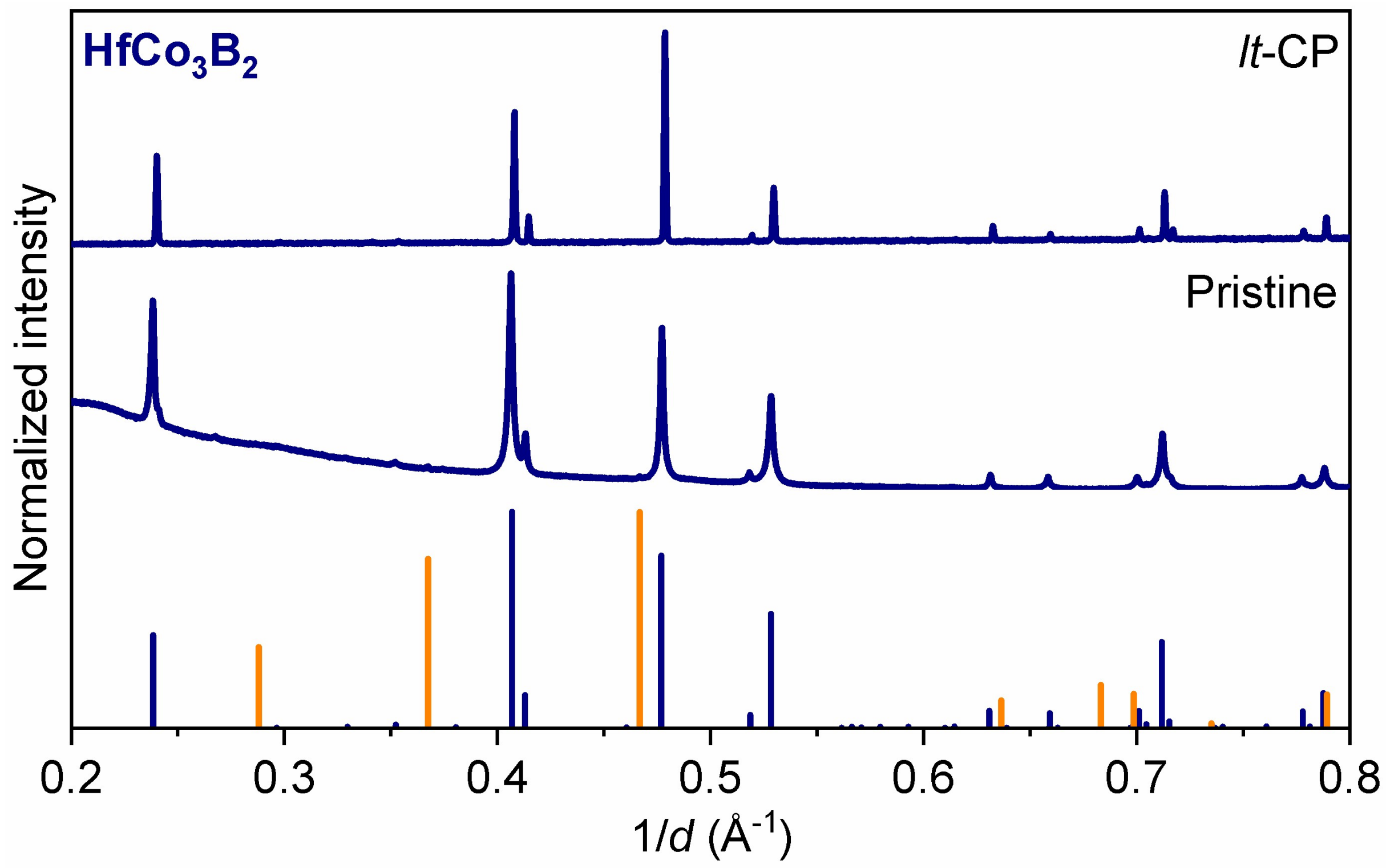

Figure S17. XRD patterns of $HfCo_3B_2$ before (transmission mode, Co $K\alpha_1$ radiation, λ = 1.78896 Å) and after *lt*-CP (reflection mode, Cu $K\alpha_1$ radiation, λ = 1.54060Å). The positions of XRD peaks for $HfCo_3B_2$.*r* (*navy lines*) and HfB_2 (*orange lines*) are shown below.

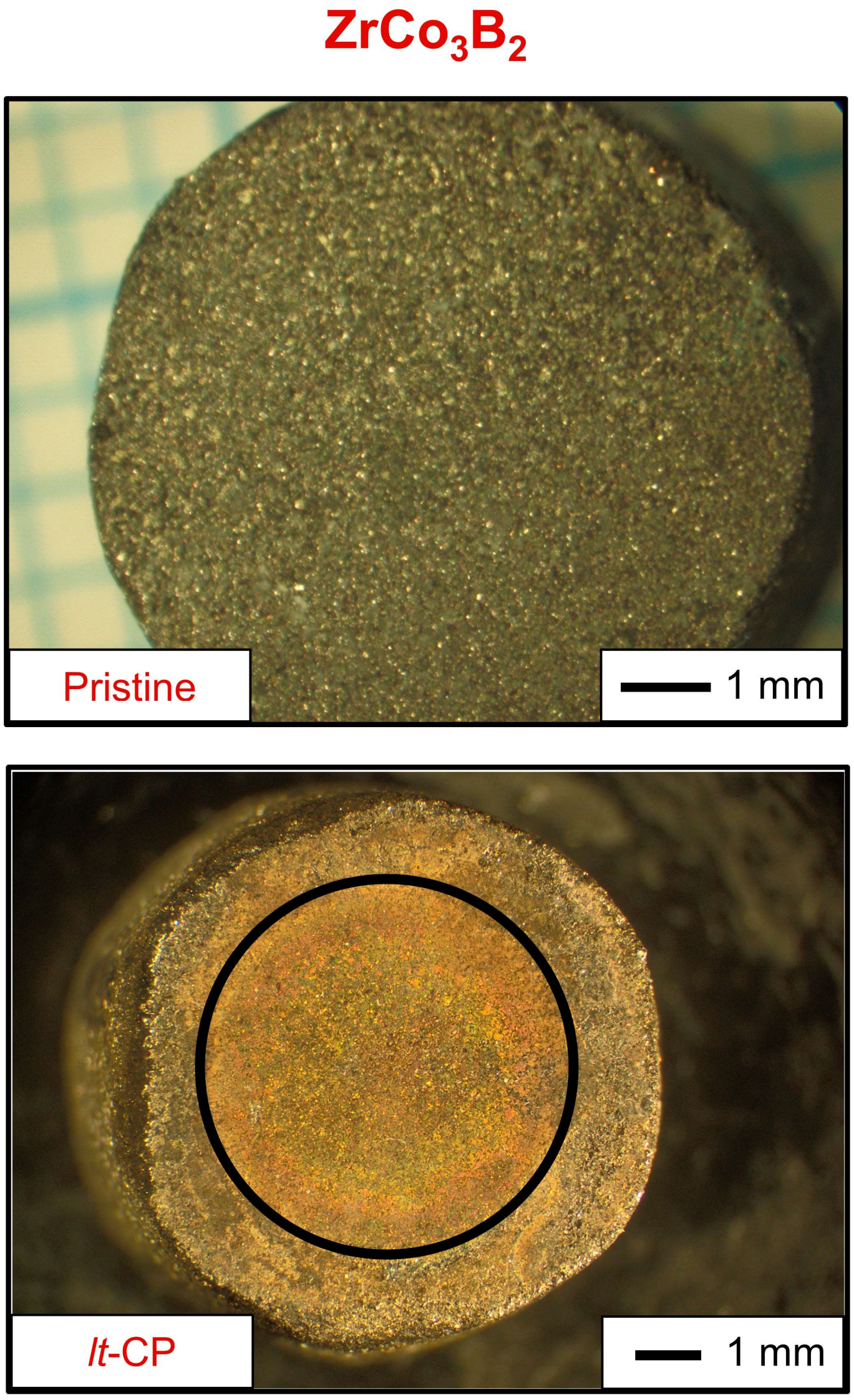

Figure S18. Light microscopy images (bright field) of $ZrCo_3B_2$ electrode before and after *lt*-CP.

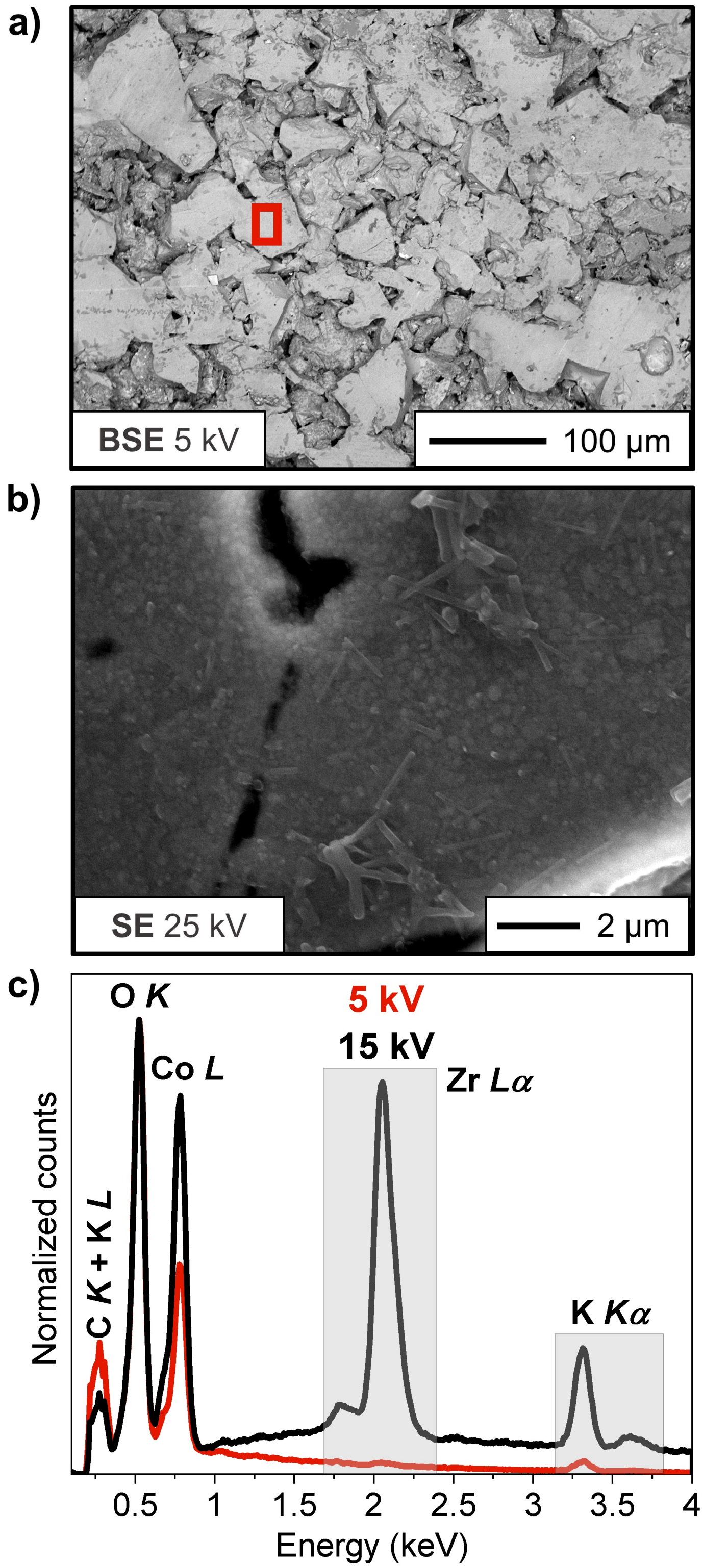

Figure S19. SEM characterization of $ZrCo_3B_2$ electrode after *lt*-CP: (*a*) BSE image (5 kV) showing the area where EDXS spectra were measured (*red rectangle*); (*b*) SE image (25 kV) of surface morphology obtained from the top of electrode; (*c*) measured EDXS spectra with different acceleration voltages (5 and 15 kV).

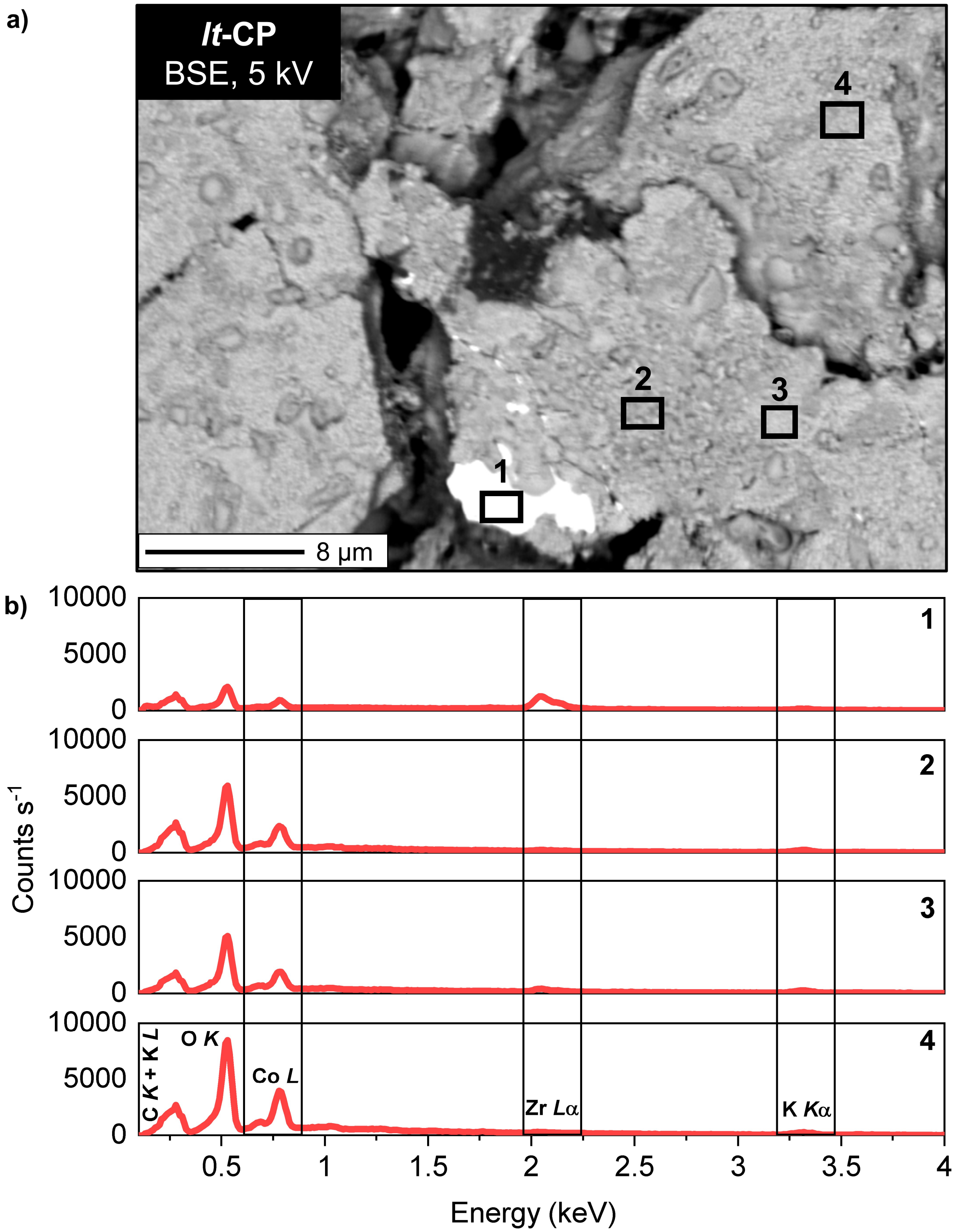

Figure S20. BSE image (*a*) and EDXS spectra (*b*), measured at acceleration voltage of 5 kV at different spots of $ZrCo_3B_2$ after *lt*-CP.

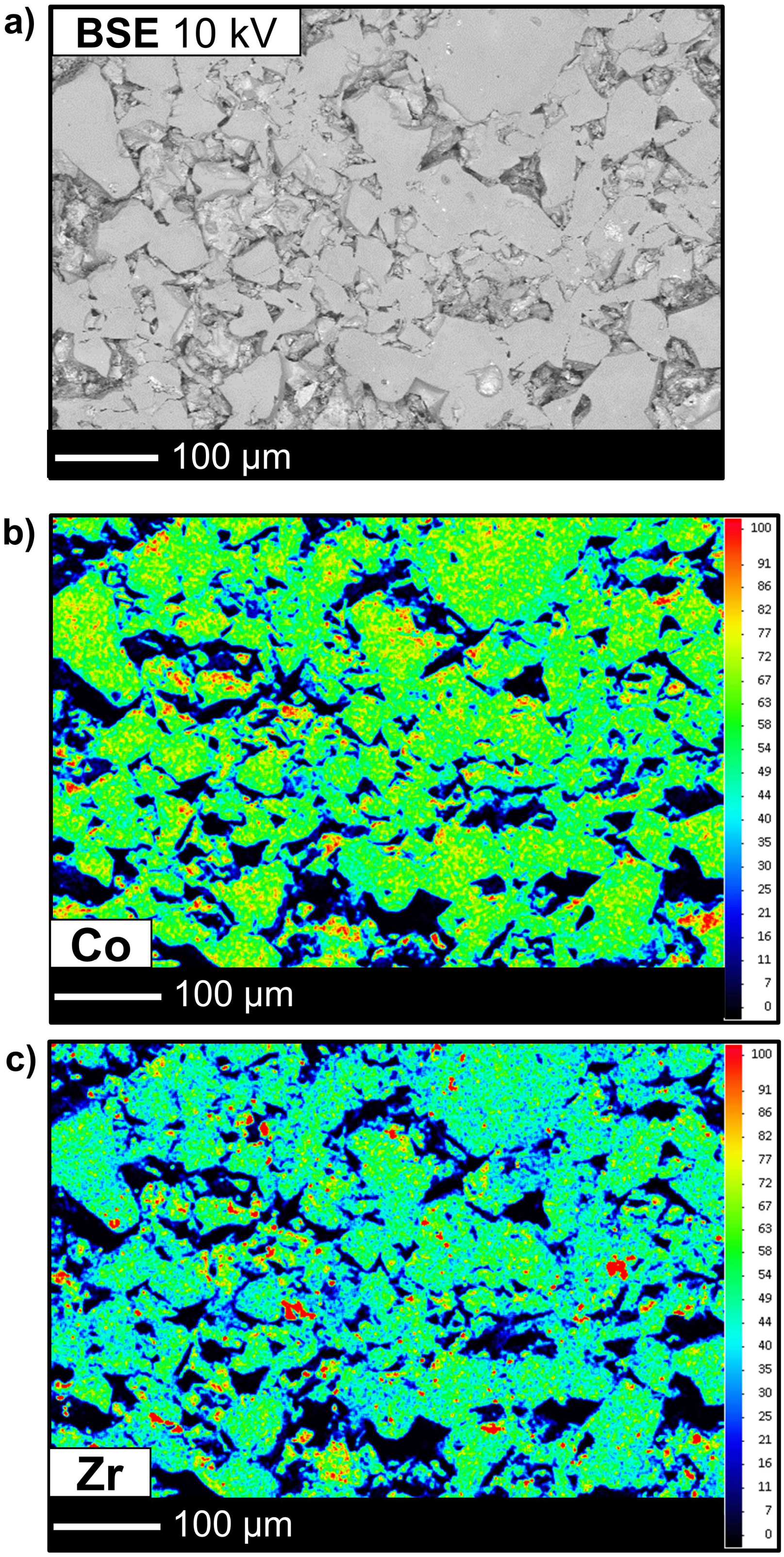

Figure S21. BSE image (acceleration voltage of 10 kV) of $ZrCo_3B_2$ electrode after *lt*-CP (*a*), supported by the Co (*b*) and Zr (*c*) elemental mappings.

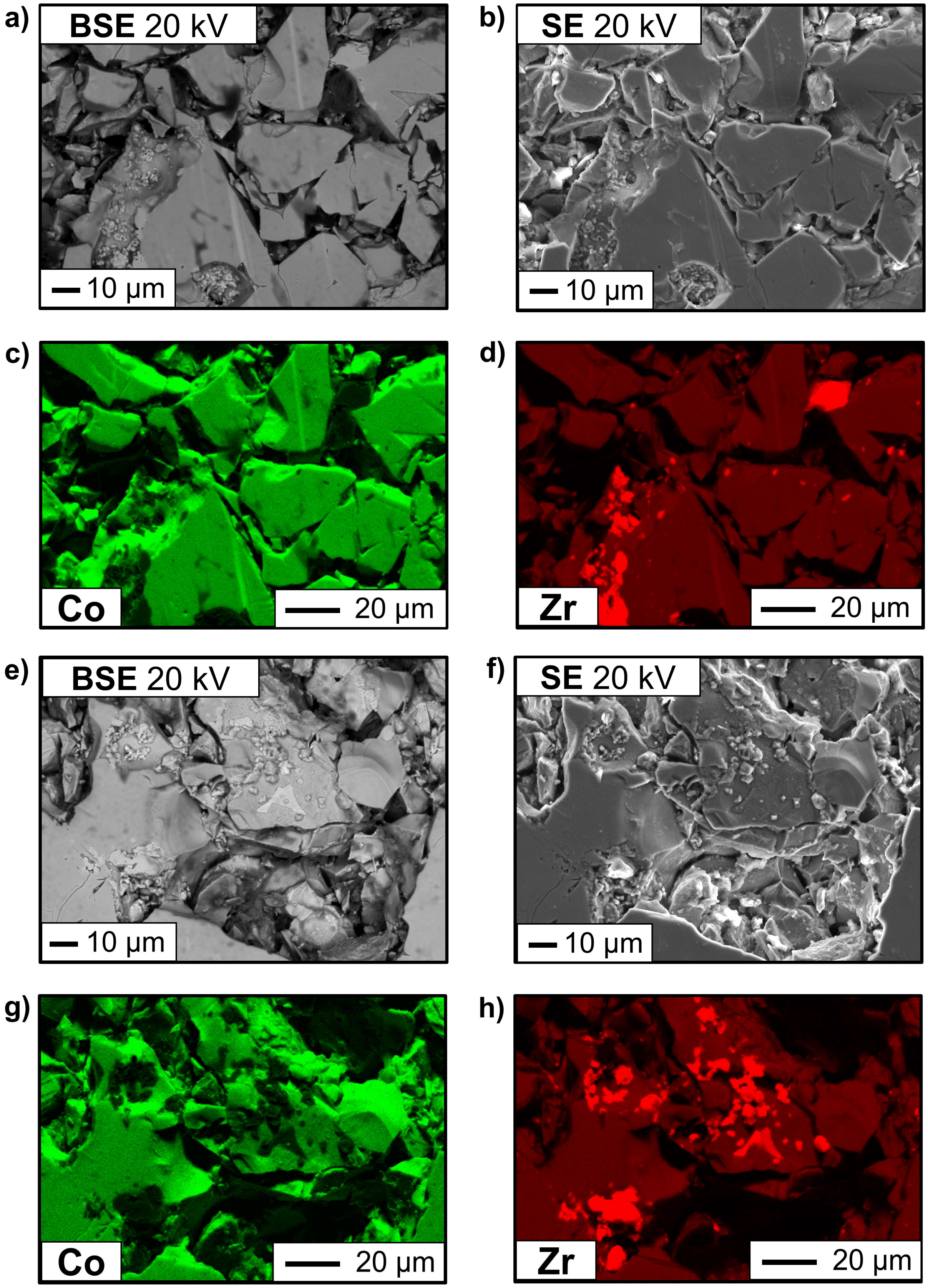

Figure S22. BSE (*a*, *e*) and SE (*b*, *f*) images, supported by Co (*c*, *g*) and Zr (*d*, *h*) elemental mappings, from two different areas of the $ZrCo_3B_2$ electrode after *lt*-CP.

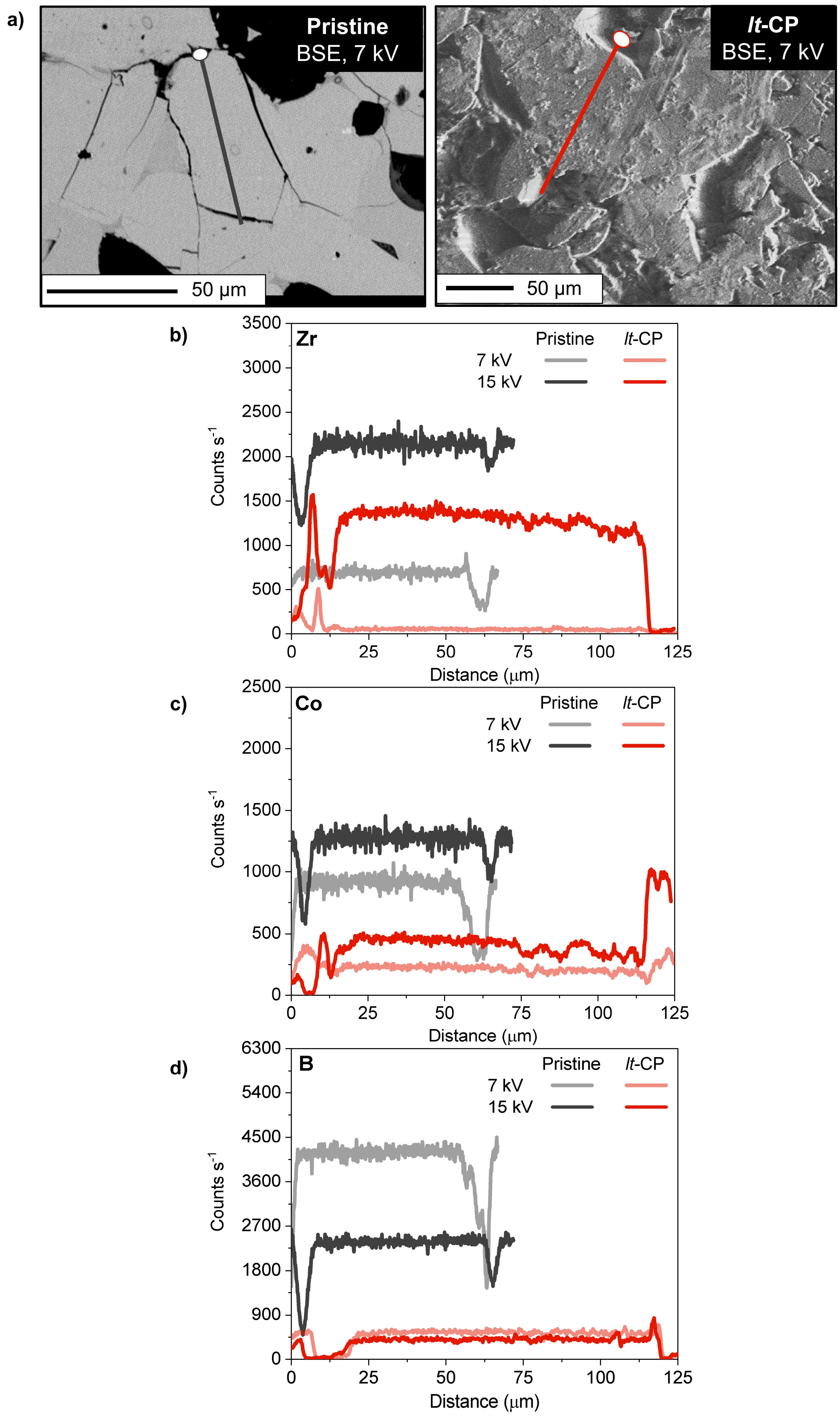

Figure S23. BSE images of pristine and after *lt*-CP (*a*) $ZrCo_3B_2$, supported by Zr (*b*), Co (*c*) and B (*d*) WDXS line scans using acceleration voltages of 7 and 15 kV.

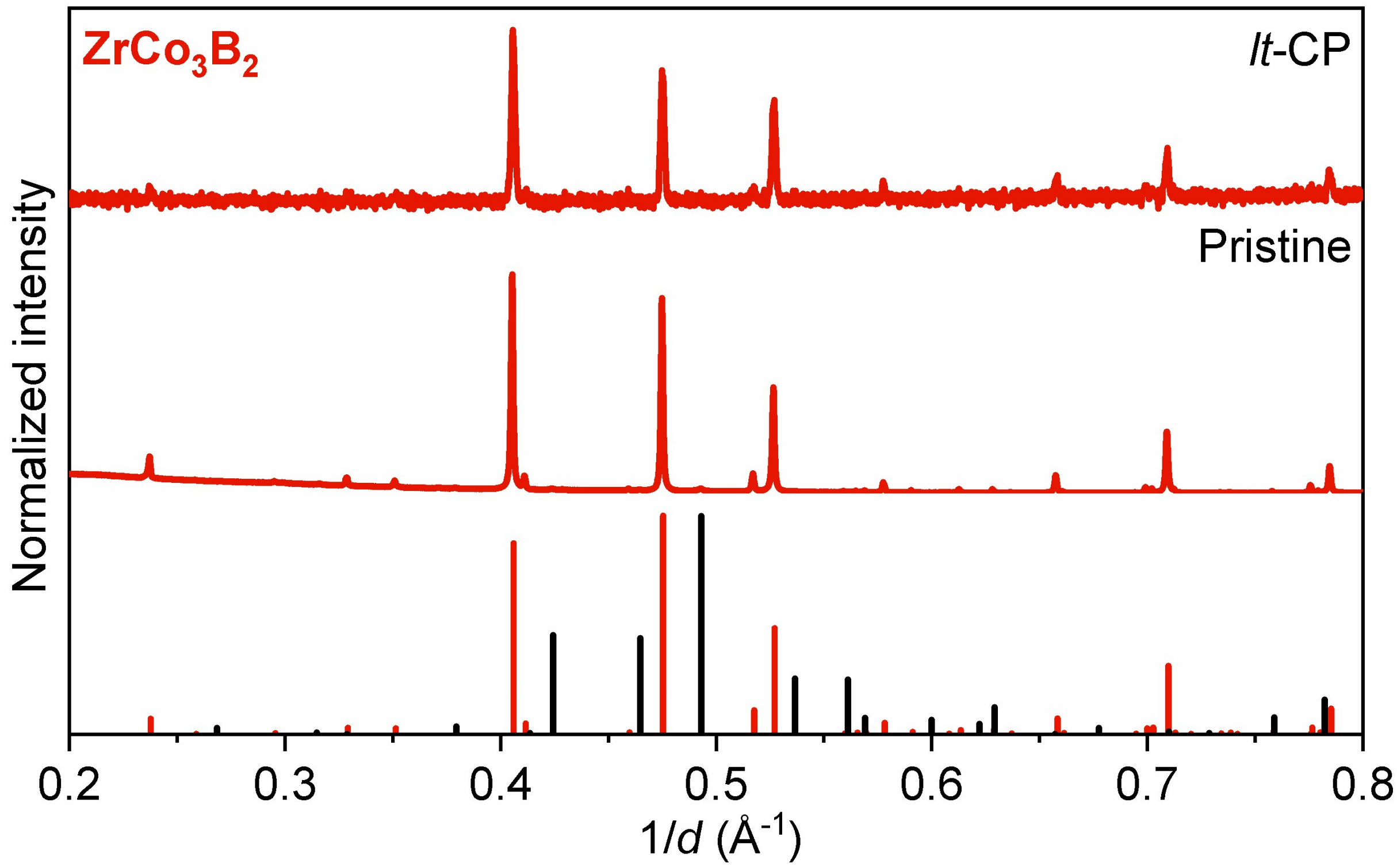

Figure S24. XRD patterns of pristine $ZrCo_3B_2$ (transmission mode, Co $K\alpha_1$ radiation, λ = 1.78896 Å) and after *lt*-CP (reflection mode, Cu $K\alpha_1$ radiation, λ = 1.54060Å). The positions of XRD peaks for $ZrCo_3B_2$.*r* (*red lines*) and $Zr_2Co_{21}B_6$ (*black lines*) are shown below.

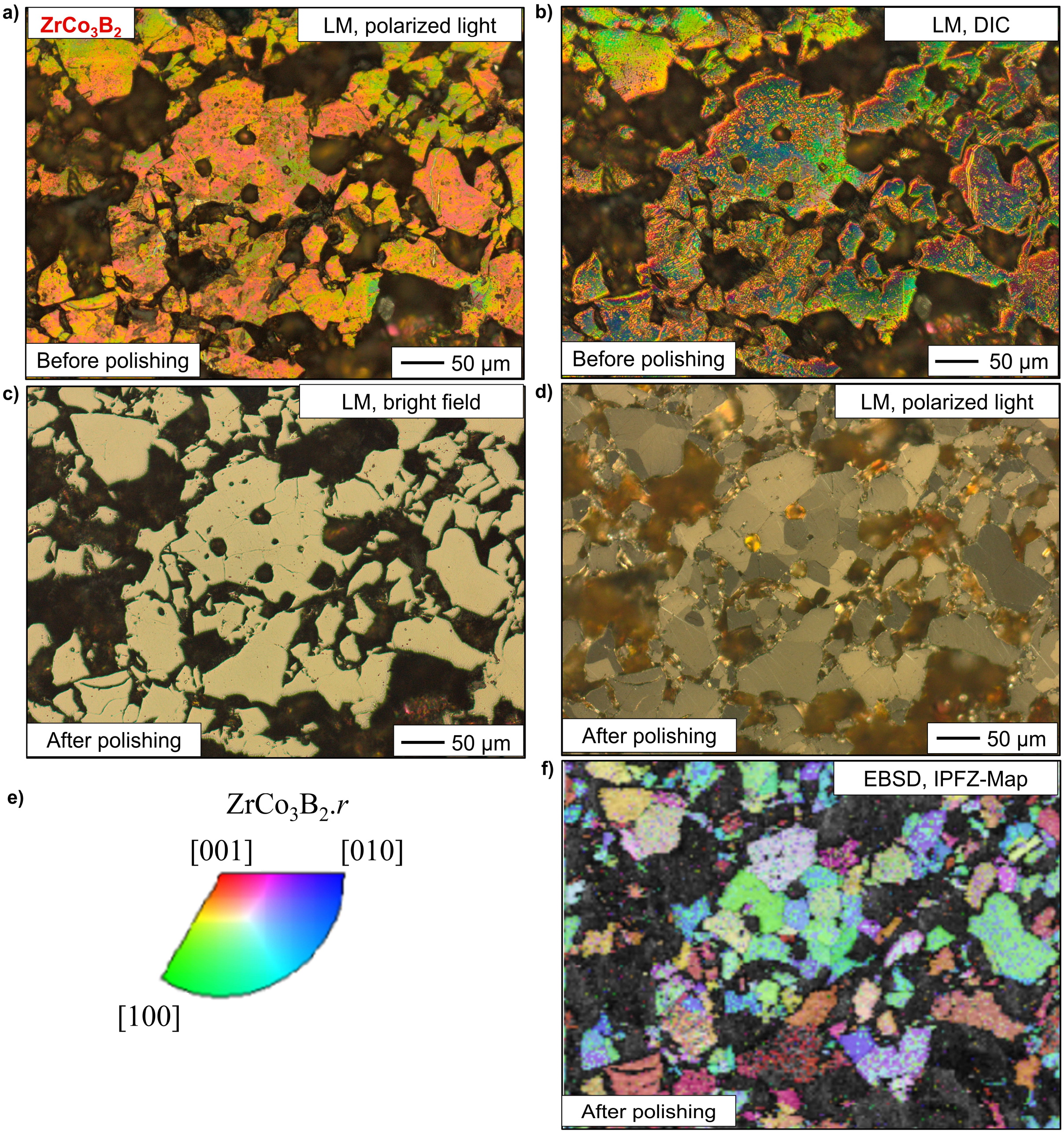

Figure S25. Light microscopy and EBSD characterization of $ZrCo_3B_2$ electrode after *lt*-CP: the LM images in the polarized light and DIC mode before (*a*, *b*) and after polishing (*c*, *d*), and EBSD IPFZ-map (*f*), supported by the color code for the orientation perpendicular to the sample surface (*e*).

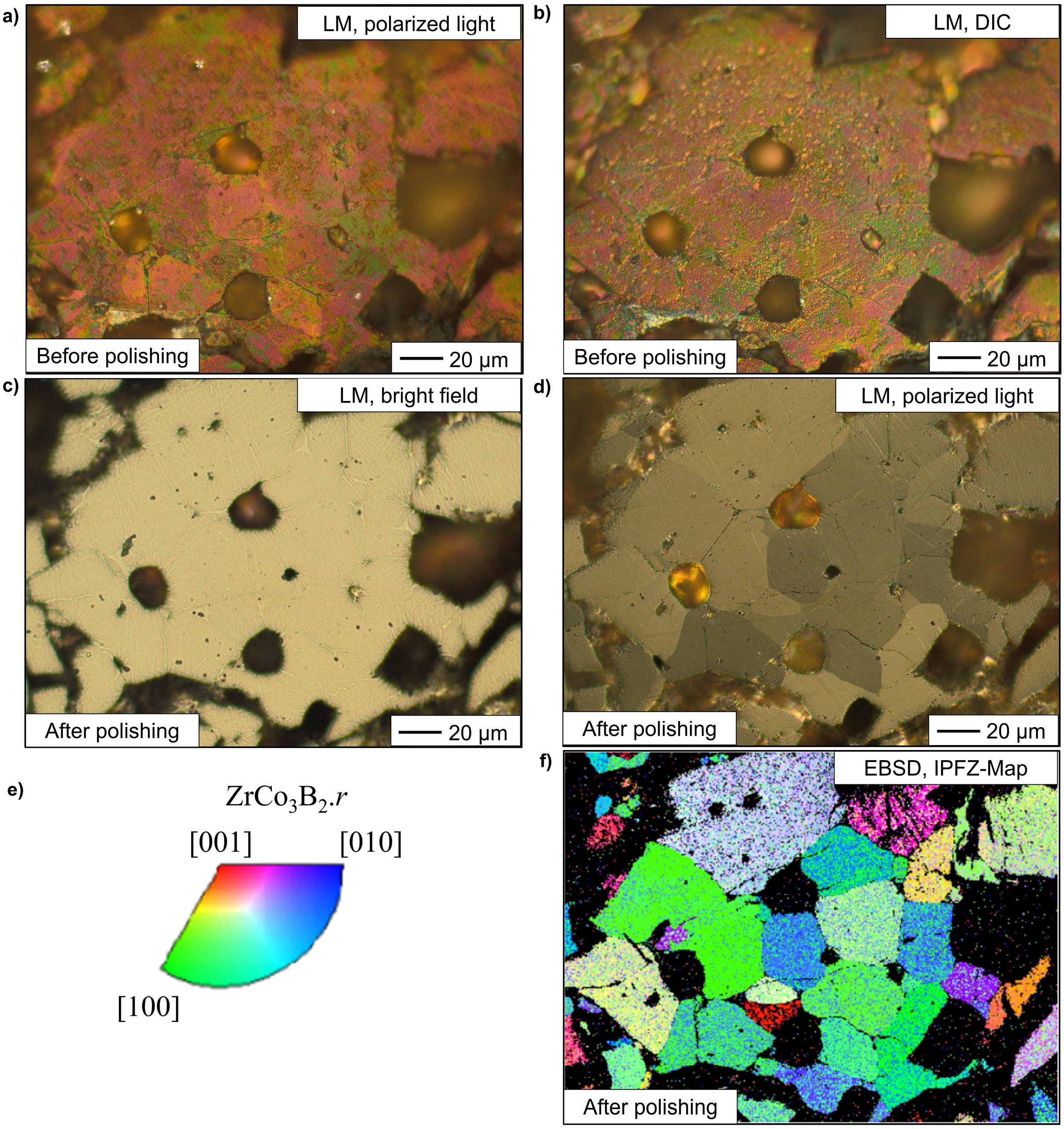

Figure S26. Light microscopy and EBSD characterization of $ZrCo_3B_2$ electrode after *lt*-CP the LM images in the polarized light and DIC mode before (*a*, *b*) and after polishing (*c*, *d*), and EBSD IPFZ-map (*f*), supported by the color code for the orientation perpendicular to the sample surface (*e*).

Table S1. OER activity of some selected Co-based electrocatalysts.

Sample	Support	Electrolyte	Overpotential at 10 mA cm^{-2} (mV)	Ref.
$ZrCo_3B_2$	–	1 M KOH	270	*This work*
$HfCo_3B_2$	–	1 M KOH	270	*This work*
$Zr_{0.8}Co_{0.2}B_2$	Glassy carbon	1 M KOH	370	[12]
$Hf_{0.8}Co_{0.2}B_2$	Glassy carbon	1 M KOH	340	[13]
Mo_2CoB_2	–	1 M KOH	290	[14]
$FeCoB_2$	Glassy carbon	1 M KOH	295	[15]
$(Ni_{0.75}Co_{0.25})_2B$	Carbon paper	1 M KOH	224	[16]
$(Co_{0.9}Ni_{0.1})_2B$	Glassy carbon	1 M KOH	371	[17]
$(Co_{0.7}Fe_{0.3})_2B$	Glassy carbon	1 M KOH	330	[18]
$LaCo_2P_2$	Ni foam	1 M NaOH	400	[19]
CoNiFe oxide	Glassy carbon	1 M KOH	230	[20]
CoNiFe oxide	Ni foam	1 M KOH	200	[20]
$(V_2O_3\text{-}CoFe_2O_4)@C/NF$	Ni foam	1 M KOH	226	[21]
$Ni_xCo_yO_4$ (x/y = ¼)	Glassy carbon	1 M KOH	336	[22]
Co_3O_4	Glassy carbon	1 M KOH	395	[22]
$Co_3O_4@CoO$	Glassy carbon	0.5 M KOH	430	[23]
Co_3B	Glassy carbon	1 M KOH	320	[24]
Co_2B	Glassy carbon	0.1 M KOH	380	[25]
Co_2B	N-doped graphene	0.1 M KOH	360	[25]
Co_2B	Glassy carbon	1 M KOH	287	[26]
$Co_2B/CoSe_2$	$CoSe_2$/DETA	1 M KOH	320	[27]
Co-B oxide (calcinated at 300 °C)	Glassy carbon	1 M KOH	338	[28]

Table S2. Concentrations of transition metals and boron (in mg L^{-1}) in spent electrolytes after CP experiments with $ZrCo_3B_2$ and $HfCo_3B_2$ electrodes (ICP-OES data).

Experiment	$ZrCo_3B_2$			$HfCo_3B_2$		
	[Zr]	[Co]	[B]	[Hf]	[Co]	[B]
***st*-CP**	< 0.05	< 0.05	< 0.05	0.2	< 0.05	< 0.05
***lt*-CP**	< 0.05	0.3	< 0.05	0.6	< 0.05	0.2

References

[1] R. J. Meyer, E. H. E. Pietsch, A. Kotowski (Editors), *Gmelins Handbuch der Anorganischen Chemie, 8. Auflage, Zirkonium*. Gmelin Institut für Anorganische Chemie und Grenzgebiete in der Max-Planck-Gesellschaft zur Förderung der Wissenschaften, Weinheim, Germany (1958).

[2] J. F. Moulder, W. F. Stickle, P. E. Sobol, K. D. Bomben, *Handbook of X-ray Photoelectron Spectroscopy*. Physical Electronics Division, Perkin-Elmer Corporation, Minnesota, USA (1992).

[3] C. Morant, L. Galán, J. M. Sanz, "An XPS Study of the Initial Stages of Oxidation of Hafnium," *Surface and Interface Analysis* 16 (1990): 304-308, https://doi.org/10.1002/sia.740160163.

[4] L. Armelao, S. Gross, E. Tondello, A. Zattin, "HfO_2-ZrO_2 Doped Silica Thin Films by XPS," *Surface Science Spectra* 10 (2003): 157-163, https://doi.org/10.1116/11.20040402.

[5] D. J. Joyner, D. M. Hercules, "Chemical Bonding and Electronic Structure of B_2O_3, H_3BO_3, and BN: An ESCA, Auger, SIMS, and SXS Study," *The Journal of Chemical Physics* 72 (1980): 1095-1108, https://doi.org/10.1063/1.439251.

[6] M. Hassel, H. J. Freund, "High Resolution XPS Study of Thin CoO(111) Film Grown on Co(0001)," *Surface Science Spectra* 4 (1996): 273-278, https://doi.org/10.1116/1.1247797.

[7] K. M. Cole, D. W. Kirk, S. T. Thorpe, "$Co(OH)_2$ Powder Characterized by X-ray Photoelectron Spectroscopy (XPS) and Ultraviolet Photoelectron Spectroscopy (UPS)," *Surface Science Spectra* 27 (2020): 024013, https://doi.org/10.1116/6.0000318.

[8] L. Armelao, D. Barreca, S. Gross, E. Tondello, "Sol-Gel and CVD Co_3O_4 Thin Films Characterized by XPS," *Surface Science Spectra* 8 (2001): 14-23, https://doi.org/10.1116/11.20010601.

[9] S. Sinha, S. Badrinarayanan, A. P. B. Sinha, "Intercalation of Oxygen with $Zr_{76}Fe_{24}$ Metglass: An X-ray Photoelectron Spectroscopy Study," *Journal of the Less-Common Metals* 125 (1986): 85-95, https://doi.org/10.1016/0022-5088(86)90082-2.

[10] L. Kumar, D. D. Sarma, S. Krummacher, "XPS Study of the Room Temperature Surface Oxidation of Zirconium and its Binary Alloys with Tin, Chromium and Iron," *Applied Surface Science* 32 (1988): 309-319, https://doi.org/10.1016/0169-4332(88)90016-5.

[11] M. McEntee, G. W. Peterson, A. Balboa, I. Iordanov, R. B. Balow, P. E. Pehrsson, "Surface Chemistry of Sulfur Dioxide on $Zr(OH)_4$ Powder: The Role of Water," *The Journal of Physical Chemistry C* 123 (2019): 17205-17213, https://doi.org/10.1021/acs.jpcc.9b00790.

[12] B. Mete, N. S. Peighambardoust, S. Aydin, E. Sadeghi, U. Aydemir, "Metal-Substituted Zirconium Diboride ($Zr_{1-x}TM_xB_2$; *TM* = Ni, Co, and Fe) as Low-Cost and High-Performance Bifunctional Electrocatalyst for Water Splitting," *Electrochimica Acta* 389 (2021): 138789, https://doi.org/10.1016/j.electacta.2021.138789.

[13] N. S. Peighambardoust, E. Sadeghi, B. Mete, M. B. Yagci, U. Aydemir, "Evaluating Electrocatalytic Activity of Metal-Substituted Hafnium Diboride ($Hf_{1-x}TM_xB_2$; *TM* = Ni and Co) Toward Water Splitting," *Journal of Alloys and Compounds* 5 (2022): 164148, https://doi.org/10.1016/j.jallcom.2022.164148.

[14] F. Aras, U. Burkhardt, A. Ormeci, H. Borrmann, S. G. Altendorf, Yu. Grin, and I. Antonyshyn, "Chemical Behavior of Mo_2TMB_2 (*TM* = Fe, Co, Ni) upon the Oxygen Evolution Reaction (OER)," *ACS Materials Au* 5 (2025): 718-731, https://doi.org/10.1021/acsmaterialsau.5c00035.

[15] X. Zou, W. Zhang, X. Zhou, K. Song, X. Ge, W. Zheng, "The Surface of Metal Boride Tinted by Oxygen Evolution Reaction for Enhanced Water Electrolysis," *J. Energy Chem.* 72 (2022): 509-515, https://doi.org/10.1016/j.jechem.2022.05.039.

[16] X. Ma, K. Zhao, Y. Sun, Y. Wang, F. Yan, X. Zhang, Y. Chen, "Direct Observation of Chemical Origins in Crystalline $(Ni_xCo_{1-x})_2B$ Oxygen Evolution Electrocatalysts," *Catal. Sci. Technol.* 10 (2020): 2165-2172, https://doi.org/10.1039/d0cy00099j.

[17] J. Schuch, S. Klemenz, P. Schuldt, A. M. Zieschang, S. Dolique, P. Connor, B. Kaiser, U. I. Kramm, B. Albert, W. Jaegermann, "Efficient Oxygen Evolution Electrocatalyst by Incorporation of Nickel into Nanoscale Dicobalt Boride," *ChemCatChem* 13 (2021): 1772-1780, https://doi.org/10.1002/cctc.202002030.

[18] S. Klemenz, J. Schuch, S. Hawel, A. M. Zieschang, B. Kaiser, W. Jaegermann, B. Albert, "Synthesis of Highly Efficient Oxygen-Evolution Electrocatalyst by Incorporation of Iron into Nanoscale Cobalt Boride," *ChemSusChem* 11 (2018): 3150-3156, https://doi.org/10.1002/cssc.201801547.

[19] D. K. Mann, A. M. Díez, J. Xu, O. I. Lebedev, Y. V. Kolen'ko, M. Shatruk, "Polar Layered Intermetallic $LaCo_2P_2$ as Water Oxidation Electrocatalyst," *ACS Appl. Mater. Interfaces* 14 (2022): 14120-14128, https://doi.org/10.1021/acsami.1c19858.

[20] L. Han, L. Guo, C. Dong, C. Zhang, J. Niu, Z. Peng, Z. Zhang, "Ternary Mesoporous Cobalt-Iron-Nickel Oxide Efficiently Catalyzing Oxygen/Hydrogen Evolution Reactions and Overall Water Splitting," *Nano Res.* 12 (2019): 2281-2287, https://doi.org/10.1007/s12274-019-2389-5.

[21] H. Zhang, G. Qian, X. Chen, W. Jiang, T. Yu, Y. Wang, L. Luo, S. Yin, "V_2O_3-Decorated Spinel $CoFe_2O_4$ with Carbon-Encapsulated Mesoporous Nanosheets for Efficient Water Splitting," *ACS Sustainable Chem. Eng.* 9 (2021): 980-986, https://doi.org/10.1021/acssuschemeng.0c08477.

[22] X. Deng, S. Öztürk, C. Weidenthaler, H. Tüysüz, "Iron-Induced Activation of Ordered Mesoporous Nickel Cobalt Oxide Electrocatalyst for the Oxygen Evolution Reaction," *ACS Appl. Mater. Interfaces* 9 (2017): 21225-21233, https://doi.org/10.1021/acsami.7b02571.

[23] C. W. Tung, Y. Y. Hsu, Y. P. Shen, Y. Zheng, T. S. Chan, H. S. Sheu, Y. C. Cheng, H. M. Chen, "Reversible Adapting Layer Produces Robust Single-Crystal Electrocatalyst for Oxygen Evolution," *Nat. Commun.* 6 (2015): 8106 (9 pp), https://doi.org/10.1038/ncomms9106.

[24] A. M. Zieschang, J. D. Bocarsly, J. Schuch, C. V. Reichel, B. Kaiser, W. Jaegermann, R. Seshadri, B. Albert, "Magnetic and Electrocatalytic Properties of Nanoscale Cobalt Boride, Co_3B," *Inorg. Chem.* 58 (2019): 16609-16617, https://doi.org/10.1021/acs.inorgchem.9b02617.

[25] J. Masa, P. Weide, D. Peeters, I. Sinev, W. Xia, Z. Sun, C. Somsen, M. Muhler, W. Schuhmann, "Amorphous Cobalt Boride (Co_2B) as a Highly Efficient Nonprecious Catalysts for Electrochemical Water Splitting: Oxygen and Hydrogen Evolution," *Adv. Energy Mater.* 6 (2016): 1502313 (10 pp), https://doi.org/10.1002/aenm.201502313.

[26] X. Ma, J. Wen, S. Zhang, H. Yuan, K. Li, F. Yan, X. Zhang, Y. Chen, "Crystal Co_xB (x = 1-3) Synthesized by a Ball-Milling Method as High-Performance Electrocatalysts for the Oxygen Evolution Reaction," *ACS Sustainable Chem. Eng.* 5 (2017): 10266-10274, https://doi.org/10.1021/acssuschemeng.7b02281.

[27] Y. Guo, Z. Yao, C. Shang, E. Wang, “Amorphous Co_2B Grown on $CoSe_2$ Nanosheets as a Hybrid Catalyst for Efficient Overall Water Splitting in Alkaline Medium,” *ACS Appl. Mater. Interfaces* 9 (2017): 39312-39317, https://doi.org/10.1021/acsami.7b10605.

[28] M. Yu, C. Weidenthaler, Y. Wang, E. Budiyanto, E. O. Sahin, M. Chen, S. DeBeer, O. Rüdiger, and Harun Tüysüz, “Surface Boron Modulation on Cobalt Oxide Nanocrystals for Electrochemical Oxygen Evolution Reaction,” *Angew. Chem. Int. Ed.* 61 (2022): e202211543 (12 pp), https://doi.org/10.1002/anie.202211543.
